# Exposure of the inner mitochondrial membrane triggers apoptotic mitophagy

**DOI:** 10.1038/s41418-024-01260-2

**Published:** 2024-02-23

**Authors:** Tahnee L. Saunders, Simon P. Windley, Gediminas Gervinskas, Katherine R. Balka, Caitlin Rowe, Rachael Lane, Maximilien Tailler, Thanh Ngoc Nguyen, Georg Ramm, Michael Lazarou, Dominic De Nardo, Benjamin T. Kile, Kate McArthur

**Affiliations:** 1 Department of Anatomy and Developmental Biology, Monash Biomedicine Discovery Institute, Monash University, Melbourne, VIC Australia; 2 Ubiquitin Signalling Division, Walter and Eliza Hall Institute of Medical Research, Melbourne, VIC Australia; 3 Department of Medical Biology, The University of Melbourne, Parkville, VIC Australia; 4 Department of Biochemistry and Molecular Biology, Monash Biomedicine Discovery Institute, Monash University, Melbourne, VIC Australia; 5 Monash Ramaciotti Centre for Cryo Electron Microscopy, Monash University, Melbourne, VIC Australia; 6 Faculty of Health and Medical Sciences, University of Adelaide, Adelaide, SA Australia; 7 Present Address: Garvan Institute of Medical Research, 384 Victoria Street, Darlinghurst, NSW 2010 Australia

**Keywords:** Autophagy, Cell death and immune response, Chaperone-mediated autophagy

## Abstract

During apoptosis mediated by the intrinsic pathway, BAX/BAK triggers mitochondrial permeabilization and the release of cytochrome-*c*, followed by a dramatic remodelling of the mitochondrial network that results in mitochondrial herniation and the subsequent release of pro-inflammatory mitochondrial components. Here, we show that mitochondrial herniation and subsequent exposure of the inner mitochondrial membrane (IMM) to the cytoplasm, initiates a unique form of mitophagy to deliver these damaged organelles to lysosomes. IMM-induced mitophagy occurs independently of canonical PINK1/Parkin signalling and is driven by ubiquitination of the IMM. Our data suggest IMM-induced mitophagy is an additional safety mechanism that cells can deploy to contain damaged mitochondria. It may have particular relevance in situations where caspase activation is incomplete or inhibited, and in contexts where PINK1/Parkin-mitophagy is impaired or overwhelmed.

## Introduction

Mitochondria are membrane-bound organelles that are essential for ATP production across all eukaryotes. Since developing a pivotal endosymbiotic relationship with a larger host cell over a billion years ago, mitochondria have become a hub for cellular signalling. Healthy mitochondrial networks are maintained via a coordinated balance between biogenesis (formation of new mitochondria), dynamics (fission and fusion), mitochondrial repair (such as the mitochondrial unfolded protein response) and mitochondrial degradation pathways (including, but not limited to, mitophagy [1]), collectively termed mitochondrial quality control. This quality control is important, not only for maintaining a healthy, functional mitochondrial network [2], but for preventing unwanted damage associated molecular pattern (DAMP) signalling. Mitochondria damaged beyond repair are targeted for removal by autophagy.

The process of autophagy can be divided into five major steps: (1) Initiation, (2) Nucleation, (3) Elongation, (4) Cargo Sequestration and Completion, and (5) Lysosomal Fusion. At initiation, autophagosome formation begins with the ULK1-complex (consisting of ULK1 and ULK2, ATG13, ATG101, and FIP200), which then triggers nucleation of the phagophore by phosphorylating components of the PI3K-Beclin1-complex (consisting of class III PI3K, VPS34, p115, Beclin-1, ATG14, and AMBRA1). The ATG12-ATG5–ATG16L1 complex then functions in phagophore elongation. Next, the ATG8s, (including LC3) undergo modifications by ATG4s, ATG3, ATG7, and the ATG5 complex, resulting in the covalent attachment to phosphatidylethanolamine to form LC3-II. LC3 is often used as a marker of autophagosomes, where it functions to sculpt the growing phagophore [3] and participate in cargo recruitment [4]. The growing phagophore then encloses the contents to form a complete autophagosome, which then fuses with a lysosome (a low pH organelle containing degradative enzymes). Autophagy can be targeted to specific organelles and in the case of mitochondria, is then referred to as mitophagy.

There are multiple mitophagy pathways that are engaged in a range of physiological and pathological settings – be that the removal of mitochondria as a result of mitochondrial damage, or during the development of specialised cells or tissues. These mitophagy pathways can be largely divided into two categories, ubiquitin-dependent and ubiquitin-independent. The best-characterised form of mitophagy is controlled by PINK1 and Parkin. Parkin is an E3 ligase that translocates to damaged mitochondria upon stabilisation of PINK1 on the outer mitochondrial membrane (OMM) [5, 6]. There, Parkin ubiquitinates OMM proteins, thereby marking damaged mitochondria for encapsulation by autophagosomes, for delivery to lysosomes. Both PINK1 and Parkin are mutated in early-onset Parkinson’s Disease (PD) [7], highlighting the importance of mitochondrial quality control in neuronal health.

In keeping with their bacterial-origins, mitochondria harbour a number of molecules that can act as pro-inflammatory DAMPs if released upon mitochondrial damage. One example is mitochondrial DNA (mtDNA) which activates the DNA sensor cGAS, then initiating the STING signalling cascade [8, 9]. Numerous studies have suggested mis-localised mtDNA may be relevant to a wide range of immune-related disorders including SLE, sepsis, liver failure, HIV, myocardial infarction, stroke, cancer, and rheumatoid arthritis [10], and increased cell-free circulating mtDNA has been reported in PD patients harbouring *PINK1* or *PRKN* mutations [11] further implicating the importance of mitophagy to a wide range of inflammatory pathologies.

It was recently reported that mitochondrial DAMP release (including mtDNA release) occurs during apoptotic cell death [12, 13] and yet despite this, apoptosis occurs in an immunologically silent manner. In this form of programmed cell death, the pro-death proteins BAX and BAK permeabilise the OMM, allowing the release of cytochrome-*c* which then activates the apoptotic caspase cascade [14]. BAX and BAK then further oligomerise to form arcs and macropores in the OMM [15, 16], through which the inner mitochondrial membrane (IMM) herniates into the cytosol [12] and becomes permeable, thereby releasing matrix components such as mtDNA [13]. If the caspase cascade is activated, rapid demolition of the apoptotic cell prevents cytokine production in response to cytoplasmic mtDNA, however, genetic or pharmacological inhibition of apoptotic caspases triggers mtDNA-induced DAMP signalling. It is currently unclear whether mitochondrial quality control mechanisms are engaged in either setting, and if so, how such pathways are initiated and regulated.

Here, we show that during intrinsic apoptosis, herniating mitochondria are sequestered by autophagosomes and delivered to a low pH environment - presumably the lysosome – for degradation. This process occurs independent of the best-characterised modulators of mitophagy, PINK1 and Parkin, but is dependent on ubiquitination of the IMM. Without IMM-ubiquitination, delivery of herniated mitochondria to lysosomes is impaired, suggesting that IMM-induced mitophagy may be important for the removal of damaged mitochondria and potential DAMPs where apoptotic caspase function or canonical mitophagy mechanisms have been compromised.

## Results

### Herniating mitochondria are targeted for degradation during apoptosis

**Fig. 1:**
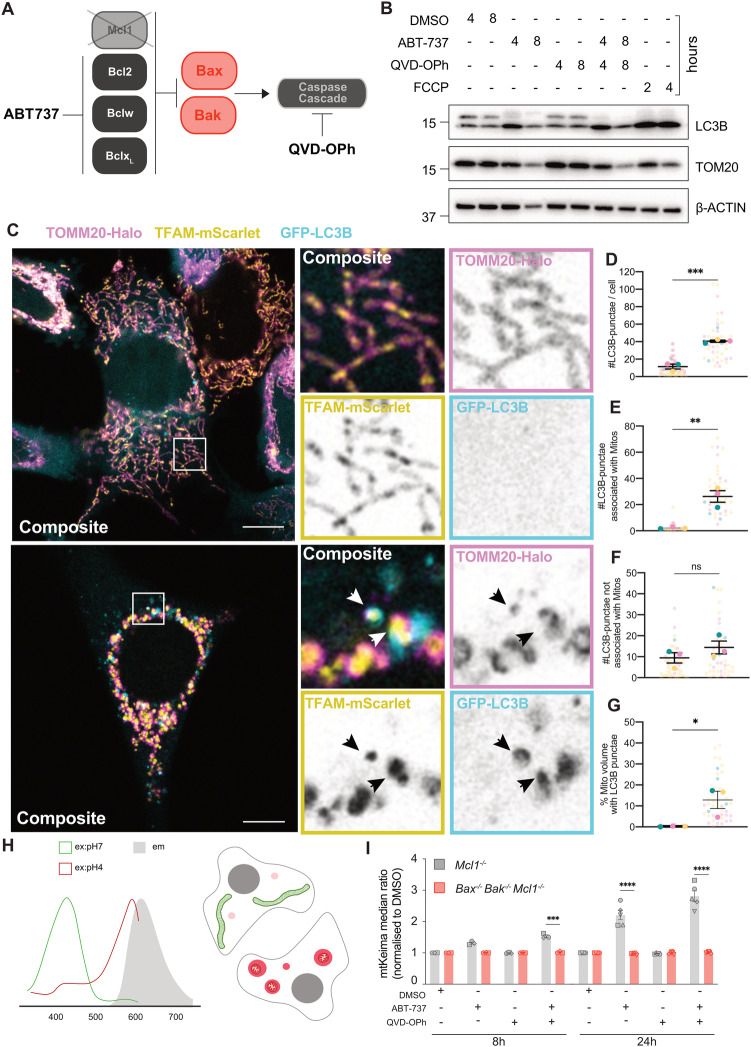
Mitophagy occurs during apoptosis. **A** Schematic of experimental model of intrinsic apoptosis induction, utilising *Mcl1*^*−/−*^ MEFs treated with the BH3 mimetic ABT-737, with or without the pan-caspase inhibitor QVD-OPh. **B** *Mcl1*^*−/−*^ MEFs were treated with ABT-737 [1 µM] ± QVD-OPh [20 µM], FCCP [30 µM], or DMSO vehicle control for indicated times and assessed by immunoblot for TOMM20, LC3B, and β-ACTIN as a loading control. Representative of n = 4 independent experiments. **C** *Mcl1*^*−/−*^ MEFs expressing TOMM20-Halo (stained with JF646, pink), TFAM-mScarlet (yellow), and GFP-LC3B (cyan) were treated for 4 h with DMSO vehicle control or ABT-737 [1 µM] and QVD-OPh [20 µM] and imaged live by Airyscan confocal imaging. Insets to the right, including single channels. Black arrows indicate herniated mitochondria associated with LC3B-puncta. **D–G** Quantification of Airyscan images of *Mcl1*^*−/−*^ MEFs expressing TOM20-Halo, TFAM-mScarlet, and GFP-LC3B shown in (**C**). **D** Number of LC3B-puncta per cell. **E** Number of LC3B-puncta associated with mitochondria. **F** Number of LC3B-puncta not associated with mitochondria. **G** The percentage of mitochondrial volume associated with LC3B puncta. Data is represented as n = 3 independent experiments where larger opaque large circles show average of each independent experiment, and smaller transparent circles represent individual cells (n = 10-20) within experiments. Circles are coloured to match individual cells with their experiment average. *p < 0.05, **p < 0.01 Paired student’s T-test. **H** Schematic of mKeima fluorescence excitation, which is dependent on pH, with the same emission. **I** Analysis of *Mcl1*^*−/−*^ or *Bax*^*−/−*^*Bak*^*−/−*^*Mcl1*^*−/−*^ MEFs expressing mtKeima, treated with DMSO, ABT-737 [1 µM] ± QVD-OPh [20 µM] for 8 h and 24 h, plotted as mean ± SEM, n = 3 independent experiments, paired data from the same experiment are represented by the same symbol shape. Statistical tests were performed as follows, **D–G** Unpaired Student’s t-test, *p < 0.05, **p < 0.01, ***p < 0.001. **I** Two-way ANOVA with Tukey correction for multiple comparisons, *p < 0.05, **p < 0.01, ****< 0.0001. Comparisons only performed between cell lines for each condition, only significant differences have been annotated on figure.

To examine if mitophagy responses occur during intrinsic apoptosis, we utilised *Mcl1*^*−/−*^ mouse embryonic fibroblasts (MEFs) treated with the BH3 mimetic ABT-737, which induces rapid-onset BAX/BAK-dependent death in these cells. Experiments were conducted in the presence or absence of the pan-caspase inhibitor QVD-OPh (Fig. 1A). Firstly, cell lysates were analysed by immunoblot at 4- and 8-h post-treatment. We observed LC3B lipidation, with either ABT-737 alone or ABT-737 and QVD-OPh in combination, as reflected by a marked increase in the lower LC3-II band (Fig. 1B). This increase was absent in *Bax*^*−/−*^ *Bak*^*−/−*^ *Mcl1*^*−/−*^ MEFs, however these cells responded normally to FCCP-induced mitophagy showing clear LC3B-lipidation (Fig. S1A, B). We then examined LC3B recruitment via live-cell imaging of *Mcl1*^*−/−*^ MEFs expressing GFP-tagged LC3B, TOMM20-Halo and TFAM-mScarlet. Vehicle-treated cells (i.e. DMSO) exhibited a diffuse cytosolic GFP-LC3B signal (Fig. 1C), while ABT-737/QVD-OPh treatment resulted in a significant increase in GFP-LC3B puncta formation (Fig. 1C, D). Of these GFP-LC3B puncta, the majority were associated with mitochondria (Fig. 1E). There was a small, but non-signicaint, increase in GRFP-LC3B puncta not associated with mitochondria upon treatment (Fig. 1F). At 4 h post-treatment, cells displayed a proportion of mitochondria associated with GFP-LC3B, averaging ~10%, with some cells displaying as much as 40% of mitochondrial volume associated with LC3B puncta (Fig. 1G). We then utilised spinning disk microscopy to examine the kinetics of these events (Movie 1). The first LC3B puncta associated with mitochondria emerged at around 20 min post-herniation. This was followed by the formation of clear GFP-LC3B vesicles completely encapsulating herniated mitochondria approximately 15 min later (Fig. S1C). In order to further monitor mitophagy during mitochondrial herniation in a more high-throughput manner, we generated cells expressing mitochondrially-targeted mKeima (referred to as mtKeima), a protein that fluoresces at different excitation wavelengths depending on the pH of its environment such that its fluorescence is distinguishable when in the low pH environment of the lysosome [17] (Fig. 1H). Consistent with our LC3B data, treatment of *Mcl1*^*−/−*^ mtKeima MEFs with ABT-737 both with or without QVD-OPh induced the delivery of mitochondria to a low pH environment (Fig. 1I). Importantly this response was dependent on BAX and BAK, as the mtKeima shift was abrogated in *Bax*^*−/−*^ *Bak*^*−/−*^ *Mcl1*^*−/−*^ MEFs (Fig. S1D, E), and was not indicative of a loss of mitophagic-capacity since *Bax*^*−/−*^ *Bak*^*−/−*^ *Mcl1*^*−/−*^ MEFs could still initiate mitophagy after DFP treatment (Fig. S1F, G). Taken together these data demonstrate that during apoptosis, herniating mitochondria are sequestered to the low pH environment of lysosomes.

### Mitochondrial sequestration during apoptosis occurs independently of PINK1/Parkin mitophagy and STING-induced autophagy

**Fig. 2:**
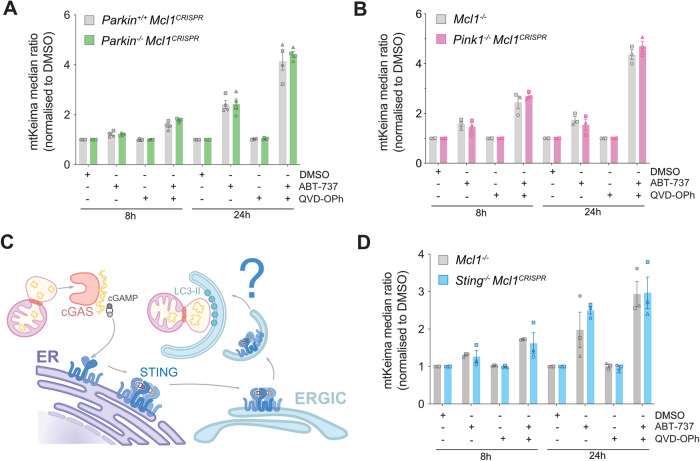
Mitophagy during apoptosis occurs independent of PINK1/Parkin mitophagy and STING-induced autophagy. Flow cytometry ratio analysis of *Mcl1*^*−/−*^ MEFs expressing mtKeima, lacking either Parkin, PINK1, or STING, and treated with DMSO, ABT-737 [1 µM] ± QVD-OPh [20 µM] for 8 h or 24 h. Data representation of mtKeima median ratio change, normalised to DMSO (**A**) *Parkin*^*+/+*^*Mcl1*^*CRISPR*^ and *Parkin*^*−/−*^*Mcl1*^*CRISPR*^ (**B**) *Mcl1*^*−/−*^ and *Pink1*^*−/−*^*Mcl1*^*CRISPR*^. **C** Schematic representation of how mtDNA release may activate STING-induced autophagy. **D** *Mcl1*^*−/−*^ and *Sting*^*−/−*^*Mcl1*^*CRISPR*^, data representation of mtKeima median ratio change, normalised to DMSO. Bars represent mean ± SEM, *n* = 3 independent experiments, paired data from the same experiment are represented by the same symbol shape.

To date, the best-characterised mitophagy pathway is regulated by PINK1 and Parkin, thus we first investigated the role of these two proteins in apoptotic mitophagy. Interestingly, MEFs lacking either Parkin (Fig. S2A) or PINK1 (Fig. S2B) were both able to undergo apoptotic mitophagy similarly to their respective WT controls (Figs. 2A, B, S2C, D). We then tested whether receptor-mediated mitophagy driven by FUNDC1 was responsible for apoptotic mitophagy, however *Fundc1*^*−/−*^ MEFs showed no change in mtKeima shift in comparison to their WT counterparts (Fig. S2E–G). Given that cGAS-STING activation is known to occur downstream of mtDNA release, combined with the suggestion that STING activation may result in autophagosome formation independent of its known role in inflammatory signalling [18] (Fig. 2C), we next generated *Sting*^*−/−*^ *Mcl1*^*CRISPR−/−*^ MEFs expressing mtKeima (Fig. S2H) and subjected them to ABT-737 and QVD-OPh treatment. The deletion of STING did not affect the mtKeima ratio shift (Figs. 2D, S2I) indicating that STING is dispensable for the mitochondrial sequestration induced by mitochondrial herniation.

### Sequestration of herniating mitochondria is partially dependent on canonical autophagy formation machinery

**Fig. 3:**
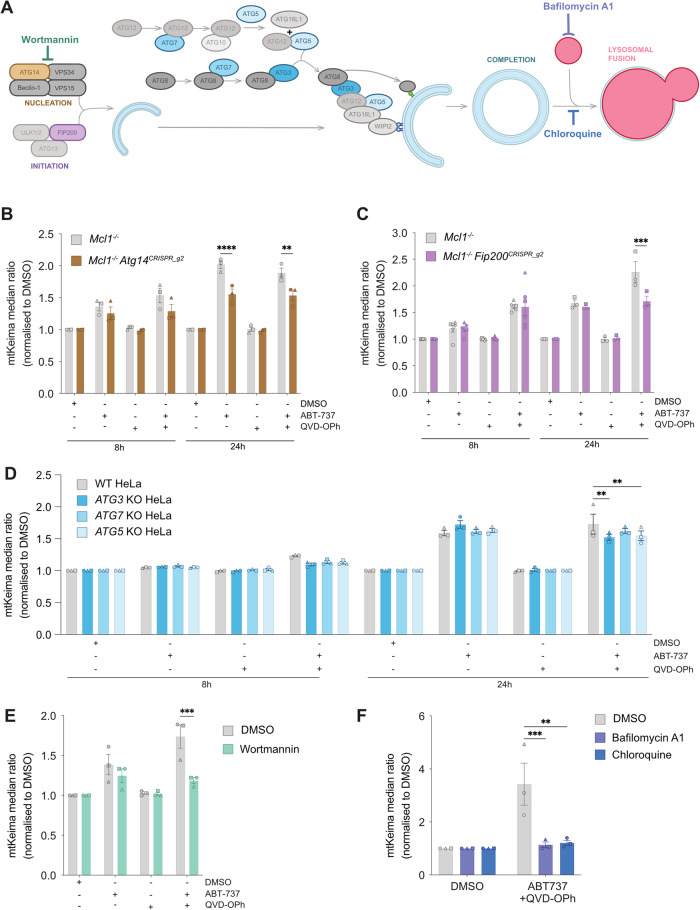
Mitophagy during apoptosis utilises canonical autophagosome formation mechanisms. **A** Schematic of autophagosome formation steps, and involved machinery, as well as pharmacological inhibitors of this process. Flow cytometry ratio analysis of *Mcl1*^*−/−*^ MEFs expressing mtKeima, lacking either (**B**) ATG14 or (**C**) FIP200, and treated with DMSO, ABT-737 [1 µM] ± QVD-OPh [20 µM] for 8 h or 24 h. **D** Flow cytometry ratio analysis of WT HeLas expressing mtKeima and lacking either ATG3, ATG7, or ATG5, and treated with DMSO, ABT-737 [1 µM] ± QVD-OPh [20 µM] for 8 h or 24 h. Flow cytometry ratio analysis of *Mcl1*^*−/−*^ MEFs expressing mtKeima, treated with DMSO, ABT-737 [1 µM] ± QVD-OPh [20 µM] in combination with (**E**) Wortmannin [1 µM] for 4 h, or (**F**) Bafilomycin A1 [25 nM] or Chloroquine [100 µM] for 16 h. Data is median ratio after treatment, normalised to DMSO control. Data are presented as mean ± SEM, n = 3 independent experiments, with paired data from the same experiment represented by the same symbol shape. Statistical tests were performed as follows, **B–D** Two-way ANOVA with Tukey correction for multiple comparisons, *p < 0.05, **p < 0.01, ****p < 0.0001, **E** Unpaired Student’s t-test, *p < 0.05 **F** Ordinary One-way ANOVA with Tukey correction for multiple comparisons, *p < 0.05.

Given the independence of apoptotic mitophagy from Parkin, PINK1, and STING, we next sought to dissect the requirement of known autophagy proteins (Fig. 3A). We began with a genetic approach, and examined the involvement of the canonical autophagosome initiation machinery proteins, ATG14 and FIP200. To this end, we generated *Mcl1*^*−/−*^ *Atg14*^*−/−*^ and *Mcl1*^*−/−*^ *Fip200*^*−/−*^ MEFs by CRISPR/Cas9 gene targeting utilising two sgRNA guides. Loss of ATG14 and FIP200 was confirmed by immunoblot, and functional assessment during starvation or DFP treatment (Fig. S3A–H) noting that *Mcl1*^*−/−*^ *Fip200*^*−/−*^ MEFs displayed low – albeit greatly reduced – levels of FIP200. Deficiency in ATG14 reduced the mtKeima shift in both knockout lines, but could not completely abrogate it (Figs. 3B and S3I, J). In contrast, reduction in FIP200 had no effect on mtKeima shift at 8 h of treatment, but exhibited a partial abrogation at 24 h (Figs. 3C and S3K, L), albeit at differing degrees between the two independent cell lines assessed. We next assessed the contribution of the ATG8 conjugation machinery, using *ATG3*^*−/−*^, *ATG7*^*−/−*^ and *ATG5*^*−/−*^ HeLa cell lines (Fig. S4A–G). Loss of ATG3 and ATG5, but not ATG7, caused a significant decrease in the mtKeima shift (Figs. 3D and S4H). Akin to the *Atg14*^*−/−*^ and *Fip200*^*−/−*^ lines, the reduction of mtKeima shift in *ATG3*^*−/−*^ and *ATG5*^*−/−*^ HeLa cells was only partial. To complement our genetic studies, we also tested well-known autophagy inhibitors of autophagosome formation (Wortmannin) or autophagosome-lysosomal fusion (BafilomycinA1 & Chloroquine) (Figs. 3E, F and S4I–J). In line with our *Atg14*^*−/−*^ data, the function of the PI3K complex contributed to mitochondrial sequestration during apoptosis, since Wortmannin treatment completely inhibited the mtKeima ratio shift after cotreatment with QVD-OPh and ABT-737 (Fig. 3E). Bafilomycin A1 and Chloroquine were also able to inhibit mtKeima ratio shift after cotreatment with QVD-OPh and ABT-737 (Fig. 3F). Taken together, these data suggest that the core canonical autophagy machinery is partially contributing to delivery of herniating mitochondria to lysosomes, reflective of either the redundancy inherent to autophagy pathways [19], or a potential alternative mechanism for mitochondrial delivery to lysosomes.

### Autophagy adaptors are recruited to sites of mitochondrial herniation during apoptosis

**Fig. 4:**
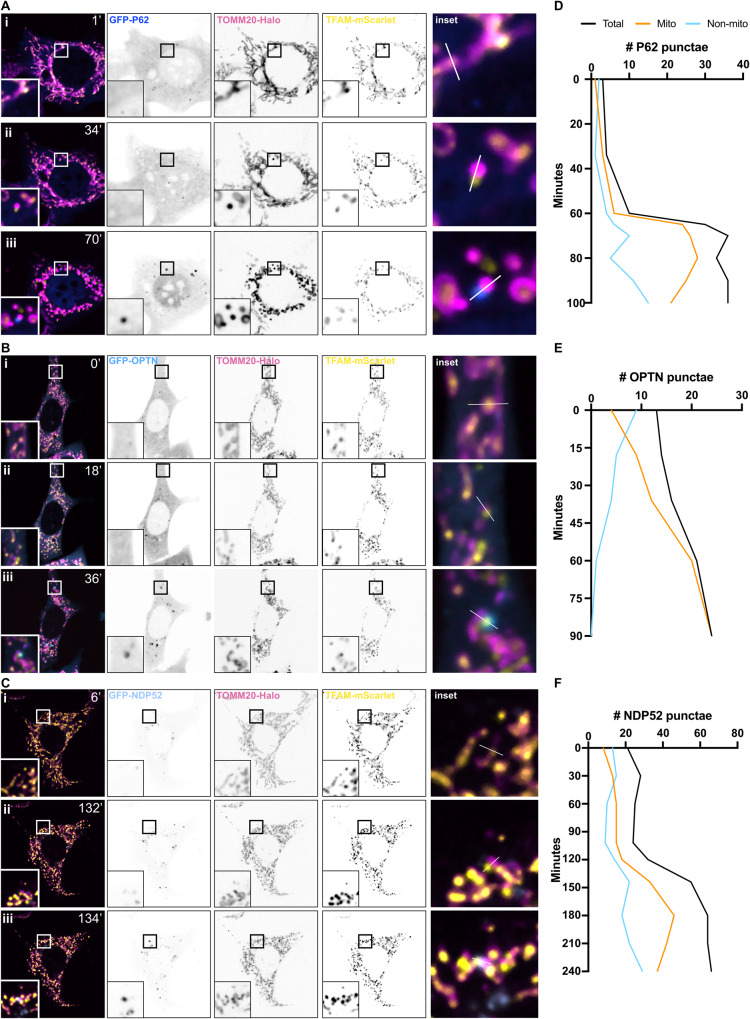
Autophagy adaptor proteins, P62, OPTN, and NDP52, are recruited to herniating mitochondria. Snapshots of live-cell spinning disk microscopy imaging of *Mcl1*^*−/−*^ MEFs expressing TOMM20-Halo (stained with JF646) (pink), TFAM-mScarlet (yellow) and, either **A** GFP-P62 (dark blue), **B** GFP-OPTN (blue), or (**C**) GFP-NDP52 (pale blue). Cells were pre-treated with QVD-OPh [20 µM] and imaging began after the addition of ABT-737 [1 µM]. Panel (i) shows timepoint zero T0, immediately after addition of ABT-737, (ii) timepoint designated T2 immediately after mitochondrial herniation, and (iii) timepoint designated T3, upon clear recruitment of autophagy adaptor protein to herniated mitochondria. **D–F** Kinetic analysis of number of GFP puncta associated with mitochondrial signals. See also Movies 2, 3 and 4.

To elucidate which proteins might be involved in marking apoptotic mitochondria for sequestration by autophagosomes, we next investigated the known autophagy adaptor proteins, P62, OPTN and NDP52. To this end, we utilised live-cell spinning disk microscopy to image MEFs co-expressing TOMM20-Halo, TFAM-mScarlet, and GFP-fusion constructs of either P62 (Fig. 4A), OPTN (Fig. 4B), or NDP52 (Fig. 4C). After induction of apoptosis, in each case we observed recruitment of the autophagy adaptor protein to herniated mitochondria, and strikingly, exclusively to the herniated side of mitochondria where the IMM was exposed (Movies 2–4). This could be visually represented by line graph (Fig. S5A–D) and kinetically quantified using Image J (Fig. 4D–F). Recent work has implicated TBK1 in both recruitment and phosphorylation of adaptor proteins such as OPTN during PINK1/Parkin mitophagy [20–23]. We therefore treated apoptotic cells with the TBK1 inhibitor BX795, or the ULK1/2 inhibitor MRT68921 and observed that the delivery of herniated mitochondria to lysosomes was significantly ablated (Fig. S5E). Together these data demonstrate autophagy adaptors are recruited to sites of mitochondrial herniation during apoptosis, potentially via a mechanism dependent on TBK1 and ULK1/2.

### Exposed IMM is ubiquitinated and acts as a signal for sequestration of herniated mitochondria

**Fig. 5:**
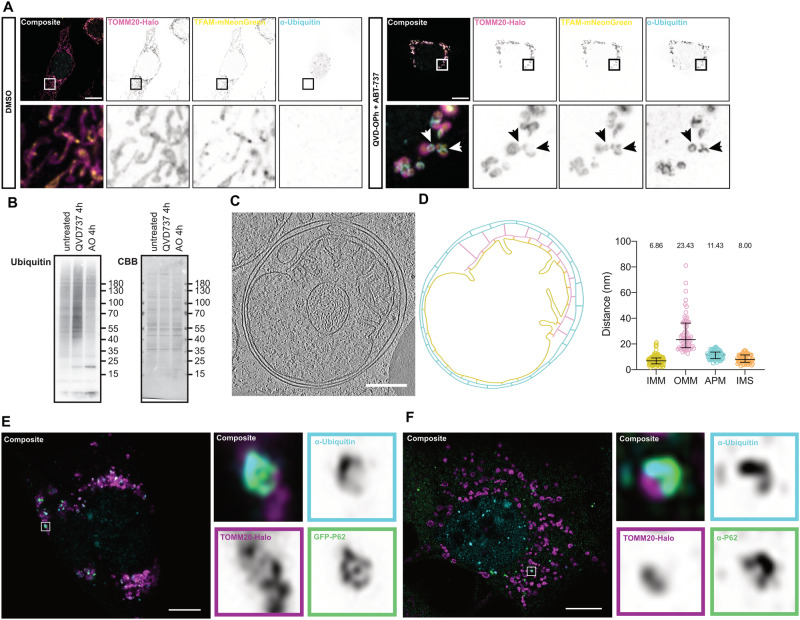
The herniated inner mitochondrial membrane is ubiquitinated, and acts as a site for formation of engulfing autophagosome. **A** Airyscan confocal imaging of *Mcl1*^*−/−*^ MEFs expressing TOMM20-Halo (stained with JF646) (pink) and TFAM-mNeonGreen (yellow) treated with either DMSO or QVD-OPh [20 µM] + ABT-737 [1 µM] for 4 h and stained for conjugated ubiquitin (light blue). **B** *Mcl1*^*−/−*^ MEFs were exposed to ABT-737 [1 µM] and QVD-OPh [20 µM] or Antimycin A [4 µM] and Oligomycin [10 µM] for 4 h, and crude mitochondrial isolates from these cells were then assessed by SDS-PAGE for Ubiquitin. CBB Coomassie brilliant blue. **C** Slice of cryo-FIB-tomogram of herniating mitochondria enveloped in an autophagosome. **D** Outline of tomogram, OMM (pink), IMM (yellow), autophagosome (light blue), including distances measured between membranes, quantified in right panel. **E** Airyscan confocal imaging of *Mcl1*^*−/−*^ MEFs expressing TOMM20-Halo (stained with JF646) (pink) and GFP-P62 (green) treated with QVD-OPh [20 µM] + ABT-737 [1 µM] for 4 h and stained for conjugated ubiquitin (light blue). Insets to the right show one herniating mitochondria. **F** Airyscan confocal imaging of *Mcl1*^*−/−*^ MEFs expressing TOMM20-Halo (stained with JF646) (pink) treated with QVD-OPh [20 µM] + ABT-737 [1 µM] for 4 h and stained for conjugated ubiquitin (light blue) and endogenous P62 (green). Insets to the right show one herniating mitochondria. **A**, **E**, **F** Scale bar is 10 µM.

Ubiquitination is a common post-translational modification that can induce myriad protein processing and signalling pathways, and plays a defining role in the induction of mitophagy. Therefore, we next examined whether herniated mitochondrial membranes are ubiquitinated during apoptosis. Immunofluorescence of ubiquitin revealed minimal background ubiquitin levels on mitochondria in control cells, whilst upon induction of apoptosis, clear mitochondrial ubiquitin staining was observed (Fig. 5A). In line with our previous data demonstrating the recruitment of autophagy adaptors to the exposed IMM, the ubiquitin staining was clearly observed in semi-circular structures surrounding TFAM-positive herniations (Figs. 5A and S6A), suggestive of direct IMM ubiquitination. Mitochondrial-specific ubiquitin levels were also assessed by SDS-PAGE of crude mitochondrial lysates. Consistent with our imaging data, a significant increase in the ubiquitin smear on isolated herniating mitochondria was observed (Figs. 5B and S6B). To further investigate this at an ultrastructural level, we utilised cryo-electron tomography in conjunction with cryo-lamella preparation by focused-ion-beam milling. We observed herniating mitochondria encapsulated within membranous structures. In the example shown, a clear double-membrane structure envelops the damaged mitochondrion (Fig. 5C), and examination of the 3D reconstructed tomograms (Movie 5) reveals this double-membrane has not yet completely fused, suggesting we captured the event mid-process. Strikingly, the IMM appeared to be closely interacting with the inner membrane of the autophagosome. Analysis of the space between membrane species within the tomogram showed a significant difference in the distance between exposed IMM and the OMM in relation to the double-membraned vesicle (Fig. 5D). This suggests that the IMM is the initiation point for autophagosome formation. Furthermore, super resolution airyscan imaging revealed both the ubiquitin and autophagy adaptor proteins were closely associated – forming distinct cup-like structures facing the IMM-side of herniated mitochondria (Figs. 5E, F and S6C, D). This was true for both exogenously expressed fluorescent-fusion proteins and antibody staining of endogenous p62 (Fig. 5F).

**Fig. 6:**
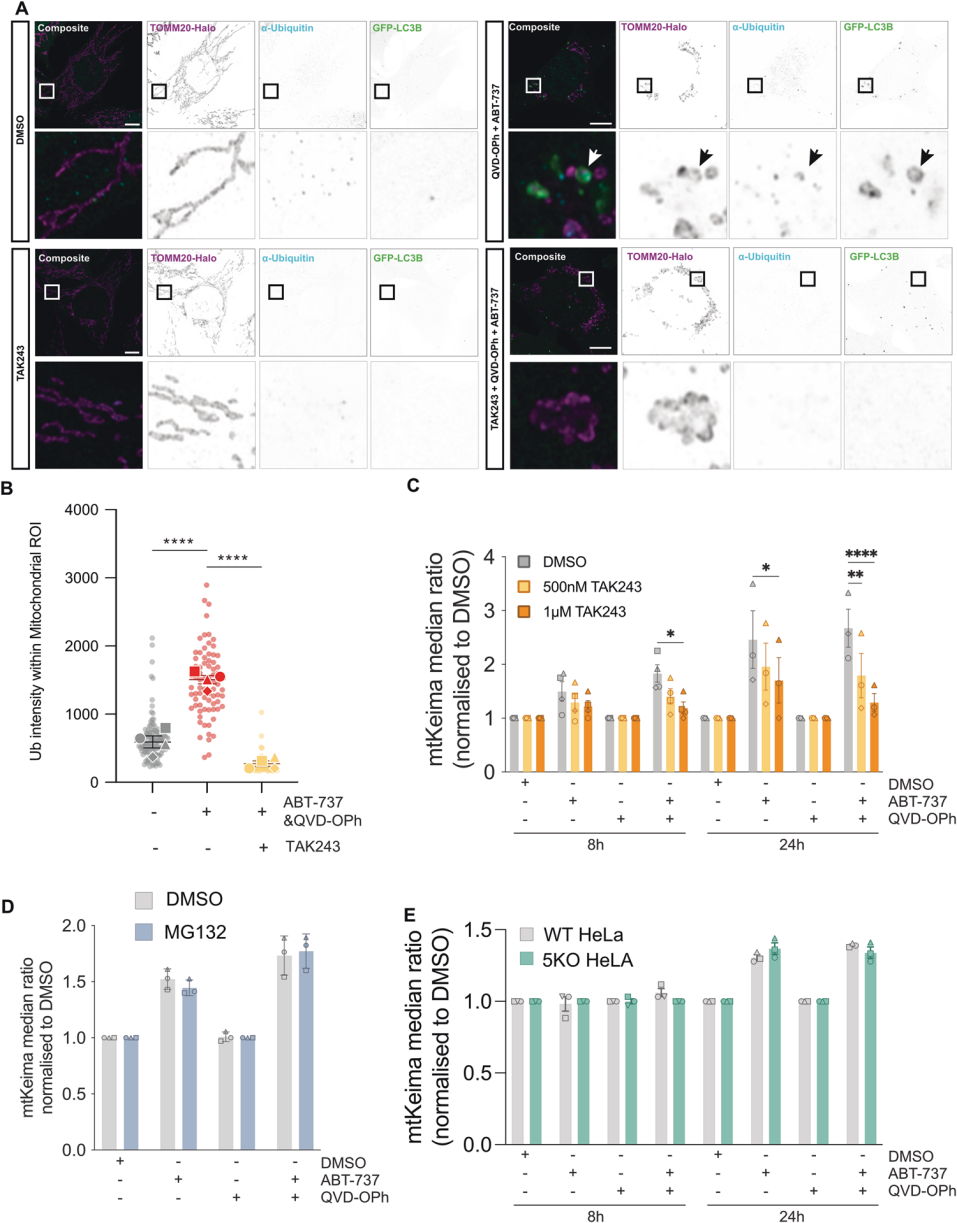
Ubiquitin of inner mitochondrial membrane is essential for apoptotic mitophagy. **A** Airyscan confocal imaging of *Mcl1*^*−/−*^ MEFs expressing TOMM20-Halo (stained with JF646) (pink) and GFP-LC3B (green) treated with either DMSO, QVD-OPh [20 µM] + ABT-737 [1 µM], TAK243 [1 µM], TAK243 [1 µM] + QVD-OPh [20 µM] + ABT-737 [1 µM], for 2 h and stained for conjugated ubiquitin (light blue). **B** Quantification of ubiquitin signal intensity present within mitochondrial region of interest. **C** Flow cytometry ratio analysis of *Mcl1*^*−/−*^ MEFs expressing mtKeima, treated for 8 h, or 24 h with DMSO, ABT-737 [1 µM] ± QVD-OPh [20 µM] in combination with increasing doses of TAK243 [500 nM and 1 µM]. **D** Flow cytometry ratio analysis of *Mcl1*^*−/−*^ MEFs expressing mtKeima, treated for 8 h, or 24 h with DMSO, ABT-737 [1 µM] ± QVD-OPh [20 µM] in combination with MG132 [1 µM]. **E** Flow cytometry ratio analysis of WT and Penta KO HeLas expressing mtKeima treated for 8 h, or 24 h with DMSO, ABT-737 [1 µM] + S63845 [2 µM] ± QVD-OPh [20 µM]. Statistical tests were performed as follows, **B** One-way ANOVA with Tukey correction for multiple comparisons, ****p < 0.0001. **C–E** Two-way ANOVA with Tukey correction for multiple comparisons, **p < 0.01, ***p < 0.001, ****p < 0.0001.

To investigate the importance of ubiquitination during induction of IMM-induced mitophagy, we utilised the small molecule inhibitor TAK243, which inhibits Ubiquitin-Activating Enzyme (UAE), the primary mammalian E1 enzyme that regulates the ubiquitin conjugation cascade [24]. Whilst treatment with Bafilomycin A1 increased the number of ubiquitin- and LC3B- associated mitochondria (Fig. S6E), the addition of TAK243 completely ablated ubiquitin immunofluorescence, and LC3B puncta were no longer detected at herniated mitochondria (Figs. 6A, B and S6F–H). Furthermore, TAK243 was also able to prevent the mtKeima shift observed during apoptosis in a dose-dependent manner (Figs. 6C and S7A). This suggests ubiquitination of herniated mitochondria is essential for their delivery to lysosomes. Given ubiquitination is often a marker for proteasomal degradation, we next investigated the impact of inhibiting the proteasome – via MG132 treatment – on our mtKeima assay. MG132 treatment was able to increase the amount of ubiquitinated proteins in cells and the accumulation of MCL1 as expected (Fig. S7B), but did not affect the delivery of mitochondria to lysosomes upon the induction of apoptosis (Figs. 6D and S7C).

Given these findings, we next applied a genetic approach to assess the necessity of a subset of ubiquitin-binding adaptors – namely TAX1BP1, NDP52, OPTN, NBR1 and p62. We expressed mtKeima in cells lacking all 5 of these adaptor proteins (“Penta KO” cells) that had been previously generated [20] and re-confirmed both their KO status and functional inability to undergo PINK1/Parkin mediated mitophagy (Fig. S7D, E). We also confirmed their ability to undergo apoptotic mitochondrial herniation (Fig. S7F). Surprisingly, mtKeima Penta KO cells were still able to deliver apoptotic mitochondria to lysosomes to equivalent levels as their wild-type counterparts (Figs. 6E and S7G), suggestive that alternate ubiquitin-binding adaptor proteins must also be involved. Together, these data suggest that ubiquitination of exposed IMM is a key initiation step for autophagosome formation and subsequent delivery of herniated mitochondria to lysosomes. Further, these data demonstrate that OPTN, p62 and NDP52 are recruited to herniated mitochondria, but are not essential to this mitophagy response.

Combined, these data suggest that during apoptosis, mitochondrial herniation results in cytosolic exposure of the IMM which is recognised and ubiquitinated, subsequently triggering mitophagy. This process does not require Parkin/PINK1, but is completely dependent on ubiquitin. Our observations that this process is only partially reliant on the canonical autophagy machinery (ATG14, FIP200, ATG3 & ATG5), suggests that there may be two pathways responsible for the delivery of apoptotic mitochondria to low pH environments – one mitophagic in nature, the other unknown. Furthermore, while recruitment of canonical autophagy adaptors (NDP52, OPTN, and p62) was clearly evident, cells lacking these autophagy adaptors were still able to deliver herniated mitochondria to lysosomes, indicating that alternate ubiquitin-binding adaptors are also involved.

This work has uncovered a unique mitophagy response to IMM-exposure hereafter termed IMM-induced mitophagy. That such a pathway or combination of pathways are employed during apoptosis reinforces the importance of containing damaged mitochondria, even during cell death.

## Discussion

We have shown that during apoptosis, mitochondrial herniation results in exposure and ubiquitination of the IMM, initiating the induction of a PINK1/Parkin-independent mitochondrial sequestration pathway. This process is BAK/BAX-dependent and occurs independently of downstream STING activation. Our images of herniating mitochondria show that the autophagy adaptors p62, OPTN, and NDP52 are recruited to ubiquitinated IMM hernias, likely serving as initiation points for autophagosome formation, although additional adaptor proteins are likely involved. Cryo-electron tomography further revealed the ultrastructure of herniated mitochondria within an autophagosome, which showed a closer association between the IMM and inner membrane of autophagosome, in comparison to the OMM. In addition, we can observe distinct electron densities, linking the IMM with the inner autophagosomal membrane, at several positions. This data supports the notion that exposure of the IMM after mitochondrial herniation results in the initiation of a specific form of apoptotic mitochondrial degradation.

In support of our findings, it has been previously reported that apoptosis and autophagy can occur in parallel to restrain mtDNA-induced IFN production via Parkin-independent mitophagy [25]. Previous studies have proposed that ATG5/7 mediated autophagy degrades *Infb1* transcripts during apoptosis. Our work demonstrates that IMM-induced mitophagy can capture herniating mitochondria, in theory preventing mtDNA release to the cytosol much earlier in the process – before any transcriptional response can be triggered. Similar images of IMM ubiquitination during a PINK1/Parkin independent form of mitophagy have recently been reported outside of apoptosis, upon treatment with the antibacterial agent actinonin [26], where p62 and OPTN recruitment to the IMM were also observed. The similarities between the mitophagy we observed during apoptosis and that induced by actinonin treatment are striking, and suggest IMM-induced mitophagy may represent a fundamental mechanism for mitophagy initiation more generally.

The idea that IMM exposure triggers a unique form of mitophagy is attractive: it is a manifestation of extreme mitochondrial damage, and presumably it is in the interest of cells to recognise such damage and respond. Several questions remain. First and foremost, the identity of the specific ligase(s) that regulates ubiquitination of exposed IMM, and the identity of its IMM substrate(s). Recent works investigating ubiquitination post-mitochondrial outer membrane permeabilization, revealed mitochondrial ligases MUL1 and MARCH5, as well as pro-apoptotic ligase XIAP, are not responsible for mitochondrial ubiquitination during apoptosis [27]. Given the high protein-lipid ratio of the IMM [28], it is likely that the unknown IMM substrates are proteinaceous, however examples of non-proteinaceous ubiquitination are becoming more numerous (including ubiquitination of LPS [29] and endosomal lipids [30]), and thus it is conceivable that an IMM lipid, such as cardiolipin, might be involved. Notably, there may also be IMM components that contribute to this novel form of mitophagy – a notion that was previously reported in the context of PINK1/Parkin mitophagy, where Prohibitin 2 (PHB2) was proposed as a mitophagy receptor [31]. Indeed, previous works suggest IMM-proteins CPOX and TRAP1 are ubiquitinated after actinonin treatment, and thus these should also be candidates.

Our data revealed that the delivery of herniated mitochondria to lysosomes during apoptosis was only partially reliant on canonical autophagy machinery, thus raising the question of what other pathways in addition to IMM-mitophagy are involved. Examples of non-autophagic mitochondrial degradation and even non-autophagic delivery of mitochondria (or mitochondrial components) to lysosomes have been documented previously.

For instance, proteasomal degradation of mitochondrial components has been shown previously to act in parallel with mitophagy – both Parkin-dependent [32] and Parkin-independent [33]. Our data did not reveal any contribution of the proteasome to the sequestration of herniated mitochondria during apoptosis, indicating that it does not play a role in IMM-mitophagy, nor can it explain any additional pathways that may be involved.

In addition, mitochondrial derived vesicles that bud from mitochondrial networks have also been implicated as a means of delivering mitochondrial components to lysosomes [34, 35], thereby compensating for a loss of canonical mitophagy [36]. These works suggest the existence of machinery capable of delivering mitochondrial constituents and membranes to lysosomes in the absence of autophagy. Further, intact – albeit damaged – mitochondria have also been reported inside endosomes in conditions where autophagy or lysosomal function has been compromised [37, 38]. In one such case, RAB7 was shown to be important for the fusion of late endosomes with lysosomes, and in the absence of RAB7 this allowed damaged mitochondria to be extruded in extracellular vesicles. Pertinent to our current study, late endosomes have are a low pH environment [39], and thus would also be detected in our mtKeima assays. Thus it is plausible that an endosomal pathway may be initiated in addition to IMM-mitophagy for the sequestration of herniated mitochondria during apoptosis.

We postulate that pathways such as IMM-mitophagy are initiated to sequester damaged mitochondria in order to curb inflammatory responses to mitochondrial components. Indeed, mitophagy has been implicated in restraining inflammation induced by damaged mitochondria in a number of different contexts. For example, PINK1/Parkin-dependent mitophagy was previously reported to be crucial for limiting mtDNA-induced inflammation in a model of Parkinson’s Disease [40] and cardiomyocytes with dysfunctional lysosomal DNAses reportedly permit mtDNA leakage and subsequent inflammatory signalling [41]. Most recently, it was also reported that when lysosomal integrity is compromised, leaking mtDNA was pro-inflammatory [42], further highlighting the anti-inflammatory functions of autophagy and the importance of constraining mitochondrial components. We posit that IMM-mitophagy is an additional avenue for containing damaged mitochondria. Thus far, our work has only focussed on IMM-mitophagy following BAK/BAX activation. Specific to this context of apoptosis, IMM-mitophagy is clearly not the only mechanism employed to limit mitochondrial induced inflammation. The apoptotic caspase cascade is the first response, demolishing the cell before inflammatory signalling can be activated, and thereby maintaining immunological silence. However, there is increasing evidence that cells can recover from sub-lethal mitochondrial damage (known as minority MOMP) and survive [43]. Whether apoptotic mitophagy – be it PINK1/Parkin- or IMM-induced – contributes to restraining mitochondrial DAMP release in such contexts or whether it aids in the recovery from minority MOMP warrants further investigation. Cell types in which inhibitors of apoptosis are highly expressed, or conversely, where key apoptotic components are lacking, may be more dependent on apoptotic mitophagy. For instance, sympathetic neurons have been reported to have high thresholds for caspase activation [44], and cardiomyocytes fail to undergo apoptosis after exogenous microinjection of cytochrome-*c* due to their reduced expression of APAF1 [45]. It may not be a coincidence that these are two cell types that exhibit heavy energy demands [46], leaving them exquisitely vulnerable to mitochondrial dysfunction and a potentially increased risk of undesirable cytochrome-*c* release. It is also important to note, that the system used in the current study was highly engineered to induce acute and specific BAX/BAK activation, and that it is likely this is very different (particularly in its kinetics and magnitude) to what happens under physiological settings. Indeed, examples of mitochondrial herniation are apparent in previously reported electron microscopy data in a variety of different non-apoptotic settings [26, 32, 47, 48]. Of note, one report observed mitochondrial herniation during an in vitro model of erythropoiesis whereby the removal of mitochondria occurs during healthy physiological conditions, in order to produce mature, mitochondria-free, red blood cells [48]. In each case, it is yet to be understood if mitophagy is employed to prevent unwanted immune activation, and if so, whether it is IMM-induced mitophagy.

We propose IMM-induced mitophagy as an additional mechanism by which cells protect against unwarranted responses elicited by damaged mitochondria. IMM-induced mitophagy may play a particularly vital role in situations where caspase activation is incomplete/absent, and PINK1/Parkin-mitophagy has either been overwhelmed or is unavailable. We posit this process may be of particular interest in the context of specific cell-types and patients with compromised PINK1/Parkin function, such as those with early-onset Parkinson’s Disease, whereby boosting IMM-induced mitophagy may provide an alternative means to clear damaged mitochondria before any release of their inflammatory constituents can trigger pathogenic neuroimmunological responses.

## Materials and methods

### Cell culture

Immortalised MEF lines were routinely maintained at 37 °C, 5% CO_2_ in in house made DME with High Glucose (DMEM (Gibco #12100) with 3.7 g/L Na2HCO3 and 0.11 g/L C_3_H_3_NaO_3_) supplemented with 10% FBS, 1% PenStrep (Thermo #15-140-122), and 100μM L-asparagine (Sigma #A4159). *Parkin*^*−/−*^ and *Pink1*^*−/−*^ MEF lines were a gift from G. Dewson. *Bax*^*−/−*^*Mcl1*^*−/−*^ and *Bax*^*−/−*^*Bak*^*−/−*^*Mcl1*^*−/−*^ lines were a gift from D. Huang. All HeLa knockout lines were a gift from M. Lazarou and were generated from ATCC HeLa S3 CCL-2.2™. To generate knockout MEFs, we used transient CRISPR/Cas9-mediated gene targeting. Targeting guide sequences were designed using the Broad CRISPick tool [49, 50], cloned into the px458 vector (containing Cas9 and GFP marker) [gift from F. Zhang [51]: Addgene plasmid 48138] and were transfected into MEFs using either Fugene 6 (Roche) at a 3:1 ratio, or Lipofectamine 2000 (Thermo Fisher Scientific) at 1.5:1 ratio. GFP-positive cells were sorted by flow cytometry (InFlux, BD) and clones derived from single cells were expanded. Knockouts were confirmed by loss of protein as assayed by immunoblot. Additionally, *Mcl1*^*CRISPR−/−*^ clones were functionally tested by assessing their sensitivity to ABT-737. The targeting guide sequences are in Supplementary Table 1.

### Genotyping

To confirm the genotypes of MEFs derived from Parkin and Pink1 mutant mice the following genotyping protocols were followed. For Pink1 knockout mice, we utilised the JAX genotyping protocol 25446: Standard PCR Assay - Pink1<tm1Shn>alternate 1, version 1.2. The common primer GCACTACAGCGAACTGCATC, was used in combination with ACTGCCACACTCAGTCCTTG (WT Rev) and GCCAGAGGCCACTTGTGTAG (Mut Rev) to generate either a band of ~500 bp to confirm Pink1 knockout status, or a band at 373 bp for WT status. For Parkin knockout mice, we utilised an in-house genotyping protocol consisting of two PCR reaction using the following primers GCCCGGTGACCATGATAG (WT Fwd); CTT CAGCGAGCTAACCTTGG (WT Rev) and GCCCGGTGACCATTGG (KO Fwd); TGAAGTGAAGGACAGCTTGACC (KO Rev) to generate either a band of ~410 bp to confirm Parkin knockout status, or a band of ~309 bp for WT status.

### Constructs and reagents

Mitochondrial imaging constructs including pMIH-TOMM20-Halo (Addgene plasmid: 111626) were generated previously [12].pMIH-TFAM-mScarlet was subcloned from pMIH-TFAM-GFP (Addgene plasmid: 113704) and PCR amplification of mScarlet from pCytERM-mScarlet-N1 (gift from D. Gadella: Addgene plasmid 85066 [52]). Autophagy protein imaging constructs were a gift from M Lazarou, including: pBMN-EGFP-LC3B, pBMN-EGFP-P62, pBMN-EGFP-OPTN, and pBMN-EGFP-NDP52. pCHAC-mtKeima was a gift from R Youle (Addgene plasmid: 72342). MEF lines stably expressing fluorescent fusion proteins were prepared by retroviral or lentiviral transduction.

Retrovirus and lentivirus were generated by transient transfection of HEK293T cells with plasmids for stable integration, along with appropriate packaging and envelope plasmids. For retrovirus production: gag-pol (packaging) and VSV-G (envelope); for third-generation lentivirus: pMDL (packaging), pRSV-REV (packaging), and VSV-G (envelope) plasmids; for second-generation lentivirus: psPAX2 (packaging), and pMD2.G (envelope) plasmids. Lipofectamine 2000 (Thermo Scientific) diluted in OptiMEM (Thermo Fisher Scientific) was used to complex plasmids into liposomes. Retrovirus and lentiviruses were harvested 24–48 h later, filtered through 0.45 µm filters and used to infect target cell lines. Cells were subsequently enriched for plasmid expression via antibiotic selection or FACS sorting.

Cell populations expressing the fluorescent fusion proteins were sorted by flow cytometry (InFlux, BD). Drug treatments used throughout the study include QVD-OPh (MedKoo Biosciences 1135695-98-5), ABT-737 (Active Biochemicals A-1002), FCCP (Sigma C2920, CAS: 370-86-5), Chloroquine (Sigma C6628, CAS: 50-63-5), Bafilomycin A1 (Sigma SML1661, CAS:88899-55-2), Wortmannin (Sigma-Aldrich W3144, CAS: 19545-26-7), DFP (Sigma 379409, CAS: 30652-11-0), BX795 (Enzo Life Sciences ENZ-CHM189-0005, CAS: 702675-74-9), MRT68921 (Selleck Chem S7949, CAS: 2070014-87-6) and TAK243 (Cayman 30108, CAS: 1450833-55-2).

### Preparation of whole-cell lysates (WCLs)

For immunoblot assays, MEFs were lysed on ice with 150 µl of 1 x Radio-immune precipitation assay (RIPA) buffer [20 mM Tris–HCl pH 7.4, 150 mM NaCl, 1 mM EDTA, 1% Triton X-100, 10% glycerol, 0.1% SDS and 0.5% deoxycholate, 5 mM NaF, 10 mM NaPPi, 1 mM Na3VO4] supplemented with 1 mM phenylmethylsulfonyl fluoride (PMSF) and 1 x cOmplete protease inhibitors (Roche Biochemicals). WCLs were clarified by centrifugation at 17,000 × *g* for 1 min through Pierce centrifuge columns (Thermo Fisher Scientific) before protein concentration was assessed by bicinchoninic acid assay (BCA; Thermo Fisher Scientific; 23227). Sample protein content was then normalised, and diluted with 4× reducing SDS-PAGE sample loading buffer [1.25% SDS, 12.5% glycerol, 62.5 mM Tris-HCl pH 6.8, 0.005% bromophenol blue, 50 mM dithiothreitol], then heated to 95 °C for 10 min.

### Isolation of crude mitochondrial lysates

Mitochondria were isolated by differential centrifugation as previously described [54]. Briefly, pelleted cells were resuspended in Isolation Buffer A (20 mM HEPES-KOH pH 7.6, 220 mM mannitol, 70 mM sucrose, 1 mM EDTA, 0.5 mM PMSF and 2 mg/mL BSA) and lysed with 20 strokes using a Dounce glass homogeniser. Lysed cells were centrifuged at 800 × *g* to pellet nuclei and intact cells. Supernatants containing mitochondria were then centrifuged at 12,000 × *g* for 10 min. The mitochondrial pellet was washed by resuspending in Isolation Buffer B (20 mM HEPES-KOH, 220 mM mannitol, 70 mM sucrose, 1 mM EDTA and 0.5 mM PMSF) and re-isolated by centrifugation at 12,000 × *g* as above. Protein concentrations were determined by BCA. Mitochondria were used immediately or aliquoted and frozen at −80 °C until required.

### Immunoblotting

WCL samples were run on either NuPAGE 4–12% Bis-Tris Protein Gels (Thermo Fisher Scientific) with MES running buffer (Thermo Fisher Scientific), or BioRad 4–20% Bis-Tris Gels (CAT#4561094 10-well, and CAT#4561096 15-well) with SDS-PAGE running buffer. Following activation of Immobilon-P polyvinyl difluoride (PVDF) membrane (Millipore Merk) in methanol, proteins were transferred to membranes using the Trans-Blot Turbo System (BioRad). Membranes were then blocked using 5% skim milk powder in Tris buffered saline (TBS) + 0.1% Tween 20 (TBST) at room temperature (RT) for 1 h before incubation overnight in primary antibodies at 4°C. Membranes were then washed 5× in TBST (5–10 min per wash) and incubated with appropriate secondary antibodies for 1–2 h at RT before membranes were again washed 5× in TBST. Chemiluminescence was detected by subjecting membranes to Immobilon Forte Western HRP substrate (Millipore Merk) before imaging using the ChemiDoc XRS+ Imaging Systems (BioRad). Images were acquired and converted to tagged image format file (TIFF) using Image Lab software (BioRad). When required, anitbodies were removed from membranes using a mild stripping buffer [1.5% glycine, 1% SDS, 0.01% Tween 20, pH 2.2] before 3x washes in TBST and re-probing with primary antibodies. Primary antibodies used for immunoblot included included mouse anti-TOMM20 (Santa Cruz Biotechnology, sc-17764), rabbit anti-LC3B (Cell Signalling Technology, 3868), mouse anti-β-Actin-HRP (Abcam, ab49900), mouse anti-Ubiquitin (Cell Signalling Technology, 3936) rabbit anti-STING (Cell Signalling Technology, 13647), rabbit anti-FIP200 (Cell Signalling Technology, 12436), rabbit anti-ATG14 (Cell Signalling Technology, 96752), mouse anti-ATP5A (Abcam, ab14748), rabbit anti-HSP60 (Cell Signalling Technology, 12165), rabbit anti-FUNDC1 (Abcam, ab224722), rabbit antI-β-Actin (Cell Signalling Technology, 4967), rabbit anti-P62 (Cell Signalling Technology, 39749), rabbit anti-P62 (Cell Signalling Technology, 23214), mouse anti-P62 (Abnova, H00008878-M01), mouse anti-ATG3 (Santa Cruz Biotechnology, sc-393660), mouse anti-ATG5 (Santa Cruz Biotechnology, sc-133158), mouse anti-ATG7 (Santa Cruz Biotechnology, sc-376212), rabbit anti-VDAC1 (a kind gift from M. Ryan), mouse anti-MCL1 (ThermoFisher Scientific, 600-401-394), rabbit anti-TAX1BP1 (Cell Signalling Technology, 5105), rabbit anti-NDP52 (Cell Signalling Technology, 9036), rabbit anti-OPTN (ProteinTech, 10837-1-AP), rabbit anti-NBR1 (Cell Signalling Technology, 20145), rabbit anti-GAPDH (Cell Signalling Technology, 21185). The HRP-conjugated secondary antibodies used were Peroxidase-AffiniPure goat anti-rabbit IgG (Jackson ImmunoResearch Labs, 111-035-003) and goat anti-mouse IgG (Thermo Scientific, A16078).

### Flow cytometry mtKeima assay

mtKeima expressing MEFs were plated in 24-well plate format, at a density of 50,000 cells/well in the morning to begin an experiment in the afternoon, or 30,000 cells/well if plated the day prior to treatment. Cells were treated at specific time-points, to allow for collection of all conditions at once. Cells were collected by taking supernatants containing floating cells, and trypsonising the remaining adherent cells. The whole-cell mixture was then centrifuged for 3 min at 300 G, media supernatant discarded, and the cell pellet washed with PBS. The cells were then spun down again and resuspended in 300 μL of FACS buffer for analysis on Fortessa X20 (BD). mKeima was excited by 488 nm and 561 nm lasers, and emission was collected using 675–705 nm and 655–685 nm filter range respectively. Data was analysed by FlowJo, by calculating a mitophagy ratio per cell, and then taking the median of this ratio from the population of cells analysed.

### Immunofluorescence

Cells were seeded into 8-well ibidi chamber slides (iBidi #80826) the day prior to treatment and fixation. When performing immunofluorescence staining on MEFs expressing TOMM20-Halo, cells were incubated with JaneliaFluor-646 HaloTag-specific dye [53], rather than staining for TOMM20. Cells were fixed by addition of freshly prepared and warmed 8% formaldehyde in cell culture media (Thermo Fisher Scientific; 16% methanol-free formaldehyde #28908) at a 1:1 ratio with cell culture media already present on cells, for 15 min at RT. Cells were then permeabilised and blocked in blocking buffer 0.15% Triton X-100, 7.5% BSA in PBS for 30 min at RT. Samples were incubated with primary antibodies diluted in blocking buffer for either 60 min at RT or at 4°C overnight. Primary antibodies used were mouse anti-ubiquitinated proteins (Sigma Aldrich, #04263), mouse anti-DNA (ThermoFisher Scientific, #61014PROGEN), rabbit anti-TOMM20 (Santa Cruz Biotechnology, #sc-11415), and anti-P62 (Cell Signalling Technology, #23214). Next, cells were washed three times with PBS and incubated with secondary antibodies diluted 1:1000 in blocking buffer for 60 min at RT. Alexa-conjugated secondary antibodies used were goat anti-rabbit IgG AlexaFlour^TM^ 647 (Thermo Scientific #A21245), and goat anti-mouse IgG AlexaFluor^TM^ plus 555 (ThermoScientific #A32727). Samples were subsequently washed 3 times with blocking buffer before nuclear staining with 300 nM DAPI (in PBS) for 10 min at RT and then a final wash using PBS.

### Confocal microscopy

Cells were plated in 8-well ibidi chamber slides (iBidi #80826) and allowed to adhere overnight. Cell treatments were performed for indicated timepoints, and were prepared in Imaging Media (Gibco DMEM, high glucose, no glutamine, no phenol red (Thermo #31-053-028), + 10% FBS, + 1% PenStrep (Thermo #15-140-122) + 100μM L-asparagine (Sigma #A4159). Airyscan confocal microscopy was performed on a Zeiss LSM 980 Confocal Microscope (containing with 405, 445, 488, 514, 561, 639 nm lasers) equipped with Airyscan 2 detector, 32 + 2 spectral GaAsP detector with two flanking PMT’s and T-PMT, as well as an environment chamber and top stage incubator maintained at 37 °C and 5% CO_2_. All Airyscan imaging utilised an 63×/1.40 NA objective. *Mcl1*^*−/−*^ MEFs expressing TOMM20-Halo, TFAM-mScarlet, GFP-LC3B, were pre-incubated with JaneliaFluor-646 HaloTag-specific dye [54] as well as indicated treatments. Images in all experimental groups were obtained using the same settings. The laser settings were determined for the first experimental replicate, saved, and re-used for repeat experiments.

### Spinning disk microscopy

*Mcl1*^*−/−*^ MEFs expressing TOMM20-Halo, TFAM-mScarlet and GFP-fusion proteins (either LC3B, p62, NDP52 or OPTN) were plated in 8-well ibidi chamber slides (iBidi #80826) and allowed to adhere overnight, before staining with JaneliaFluor-646 HaloTag-specific dye [54] prior to experiment initiation. Live spinning disk imaging was performed on the Marianas SDC system (3i) fitted with a custom stage top incubator (Oko Labs). During imaging, cells were maintained at 37 °C and 5% CO_2_, in Imaging Media (Gibco DMEM, high glucose, no glutamine, no phenol red (Thermo #31-053-028), + 10% FBS, + 1% PenStrep (Thermo #15-140-122) + 100 μM l-asparagine (Sigma #A4159)) and were imaged with Zeiss Plan-ApoChromat 63x/1.4NA oil immersion objective, with dual Prime BSI Express sCMOS (Teledyne Photometrics, Tucson, Arizona) cameras, at 4 min intervals using Slidebook 6. Post-acquisition, images were viewed in SlideBook and converted to maximum intensity projections for publication using FIJI.

### Fluorescence microscopy image analysis

FIJI was used for image analysis performed. For analysis of LC3B puncta associated with mitochondria, images were processed by background subtraction using a 2D sliding paraboloid background subtraction (50pixel radius), followed by contrast enhancement by 0.2% saturation. A manual region of interest (ROI) was then applied to each individual cell, duplicated, and then cleared from any signal outside the ROI. All three channels were then split, and the image calculator was used to combine the TOMM20 and TFAM channels to produce a whole mitochondria channel. Manual thresholding was then performed on the LC3B and mitochondria channels, and the co-localise threshold process applied. The R value and % overlap from the co-localisation threshold analysis output were plotted. In addition, the co-localisation image output was processed by the split channels function, and the overlapping channel was processed by the find edges function, and the number of particles analyses to produce the number of points where LC3B puncta associated with mitochondria.

### Cryo-FIB-milling and cryo-electron tomography

Cells were grown overnight on 200 mesh Quanti-foil gold grids that were sterilised using UV light and pre-coated with gelatin. Grids were coated by careful addition a 10 μL droplet of PBS + 0.1% gelatin at 4°C overnight. MEFs were grown on grids for 2 days before treating with indicated drug for the reported time. MEFs vitrified by plunge freezing in liquid ethane after backside blotting (frontside filter paper was replaced with parafilm or Teflon) using a Vitrobot Mark IV. Specimen grids were clipped into autoloader grid support, prior to loading into the Leica VCT500 cryo-transfer on the FEI Helios G4 UX Cryo-FIBSEM. The grid is clipped so that the cell side (the carbon side) is facing towards the flat surface of the autogrid, so that the cells are visible to the ion beams. An organo- platinum gas injection system is used to coat the surface of the grid, leaving a protective layer of material, which circumvents issues with beam-induced charging. Cryo-lamellae were milled at as low-and-angle as possible, ideally at 11°, although in some instances, cells were milled at 15°. Cryo-lamellae were thinned to approximately 200 nm thickness and grids were transferred to a FEI Titan Krios G1 Cryo-TEM. Cryo-tomograms were recorded at 2 degrees steps from ±60° to ±60° at a pixel size of 0.73 nm using an energy filter slit of 20 eV and a K2 Summit camera at a cumulative dose of about 90 electrons per Angstrom. Reconstruction was done using the IMOD Tomography package. Segmentation was performed manually using functions in FIJI, Microscopy Image Browser and Amira software packages.

### Movie 1 Herniating mitochondria are enveloped by LC3B during apoptosis

*Mcl1*^*−/−*^ MEFs expressing TOMM20-Halo (stained with JF646, pink), TFAM-mScarlet (yellow), and GFP-LC3B (cyan) were imaged utilising spinning-disk microscopy. Cells were pre-treated with QVD-OPh [20 µM] and imaging began after the addition of ABT-737 [1 µM].

### Movie 2 Autophagy adaptor P62 is recruited to herniating mitochondria

Live-cell spinning disk microscopy imaging of *Mcl1*^*−/−*^ MEFs expressing TOMM20-Halo (stained with JF646) (pink), TFAM-mScarlet (yellow) and, GFP-P62 (dark blue). Cells were pre-treated with QVD-OPh [20 µM] and imaging began after the addition of ABT-737 [1 µM].

### Movie 3 Autophagy adaptor OPTN is recruited to herniating mitochondria

Live-cell spinning disk microscopy imaging of *Mcl1*^*−/−*^ MEFs expressing TOMM20-Halo (stained with JF646) (pink), TFAM-mScarlet (yellow) and GFP-OPTN (blue). Cells were pre-treated with QVD-OPh [20 µM] and imaging began after the addition of ABT-737 [1 µM].

### Movie 4 Autophagy adaptor NDP52 is recruited to herniating mitochondria

Live-cell spinning disk microscopy imaging of *Mcl1*^*−/−*^ MEFs expressing TOMM20-Halo (stained with JF646) (pink), TFAM-mScarlet (yellow) and GFP-NDP52 (pale blue). Cells were pre-treated with QVD-OPh [20 µM] and imaging began after the addition of ABT-737 [1 µM].

### Movie 5 3D reconstructed tomogram of herniating mitochondria within double-membraned autophagosome

Cryo-FIB-tomography revealed a 3D structure of herniating mitochondria being enveloped by an autophagosome.

### Supplementary information

Supp Figure Legends

Figure S1

Figure S2

Figure S3

Figure S4

Figure S5

Figure S6

Figure S7

Movie1

Movie2

Movie3

Movie4

Movie5

WB Supplementary Figures

Supplementary Table 1

Supplementary Movie Legendes

## Data Availability

All raw data generated for this study is available from the corresponding author upon reasonable request.
