# Supplementary material for: Exposure of the inner mitochondrial membrane triggers apoptotic mitophagy
Source: Cell Death Differ. 2024 Feb 23;31(3):335–47. doi: 10.1038/s41418-024-01260-2 (PMC10923902; doi:10.1038/s41418-024-01260-2)
Supplement: Supplementary file 1 — Supp Figure Legends [file 41418_2024_1260_MOESM1_ESM.docx]

**Figure S1 Spinning Disk snapshots of LC3B associating with mitochondria after herniation.**

(**A**) *Mcl1^-/-^* and *Bax^-/-^Bak^-/-^Mcl^-/-^* MEFs were treated with ABT-737 [1µM] ± QVD-OPh [20µM], FCCP [30µM], or DMSO vehicle control for indicated times, assessed by immunoblot for TOMM20, LC3B, and β-ACTIN as a loading control. Representative of n=2 independent experiments. (**B**) LC3-II to LC3-I ratio quantified by densitometry analysis of immunoblots in Figure 1B and (**A**). (**C**) Spinning disk microscopy of *Mcl1^-/-^* MEFs expressing TOMM20-Halo (stained with JF646, pink), TFAM-mScarlet (yellow), and GFP-LC3B (cyan) were treated with QVD-OPh [20µM] and ABT-737 [500nM] and imaged at 4-minute intervals. Snapshots are shown at 20mins: just prior to mitochondrial network breakdown, 72mins: upon initial herniation events, 92mins: upon initial association with LC3B puncta, and 108mins: showing encapsulating LC3B autophagosome around a herniated mitochondrion. Data presented as maximum intensity projections. Corresponds to **Movie 1**. Data representative of n=4 experiments. (**D-G**) Analysis of *Mcl1^-/-^* or *Bax^-/-^Bak^-/-^Mcl1^-/-^* MEFs expressing mtKeima, treated with DMSO, ABT-737 [1µM] ± QVD-OPh [20µM], FCCP [30µM] or DFP [100µM]. (**D-E**) Representative flow-cytometry histograms, plotting the median ratio between ex:560nm (pH4) and ex:488nm (pH7) (**D**) shows *Mcl1^-/-^* MEFs after 8h of treatment. (**E**) shows *Mcl1^-/-^* MEFs and *Bax^-/-^Bak^-/-^Mcl1^-/-^* MEFs after 24h of treatment. (**F**) Flow cytometry ratio analysis of *Mcl1^-/-^* or *Bax^-/-^Bak^-/-^Mcl1^-/-^* MEFs expressing mtKeima, treated for 2h with FCCP [30µM]. (**G**) Flow cytometry ratio analysis of *Mcl1^-/-^* or *Bax^-/-^Bak^-/-^Mcl1^-/-^* MEFs expressing mtKeima, treated for 24h with DFP [100µM]. Plotted as mean ± SEM, n=3 independent experiments, paired data from the same experiment are represented by the same symbol shape. Statistical tests were performed as follows, (**F-G**) Two-way ANOVA with Tukey correction for multiple comparisons, ** p<0.01.

**Figure S2 Mitophagy during apoptosis occurs independent of PINK1/Parkin mitophagy, FUNDC1 receptor-mediated mitophagy and STING-induced autophagy.** (**A**) PCR of wildtype (WT) and knockout (KO) alleles of *Parkin^+/+^* and *Parkin^-/-^* MEFs, confirming genotypes. (**B**) PCR of WT and KO alleles of *Pink1* WT and *Pink1^-/-^* MEFs, confirming genotypes. (**C-D**) Representative flow-cytometry histogram plotting the ratio between ex:560nm (pH4) and ex:488nm (pH7) at 24h, for (**C**) *Parkin^+/+^Mcl1^CRISPR^* and *Parkin^-/-^Mcl1^CRISPR^* MEFs, and (**D**) *Mcl1^-/-^* and *Pink1^-/-^Mcl1^CRISPR^* MEFs. (**E**) SDS-PAGE of *Mcl1^-/-^* and *Mcl1^-/-^Fundc1*^CRISPR^ MEFs confirming loss of FUNDC1 protein. (**F**) Flow cytometry ratio analysis of *Mcl1^-/-^* and *Mcl1^-/-^Fundc1*^CRISPR^ MEFs expressing mtKeima, treated with DMSO, ABT-737 [1µM] ± QVD-OPh [20µM] for 24h. (**G**) Representative flow-cytometry histogram plotting the ratio between ex:560nm (pH4) and ex:488nm (pH7) at 24h, for *Mcl1^-/-^* and *Mcl1^-/-^Fundc1*^CRISPR^ MEFs. (**H**) SDS-PAGE of *Mcl1^-/-^* and *Sting^-/-^Mcl1^-/-^* MEFs, confirming loss of STING protein. (**I**) Representative flow-cytometry histogram plotting the ratio between ex:560nm (pH4) and ex:488nm (pH7) at 24h, for *Mcl1^-/-^* and *Sting^-/-^Mcl1^-/-^* MEFs.

**Figure S3 Knockout assessment and functional testing of MEFs lacking ATG14 or FIP200.** (**A**) Immunoblot of *Mcl1^-/-^ and Mcl1^-/-^Atg14*^CRISPR^ MEFs, confirming loss of ATG14 protein. (**B**) Immunoblot of *Mcl1^-/-^ and Mcl1^-/-^Atg14*^CRISPR^ MEFs after serum starvation, with or without bafilomycin A1 treatment. Representative of 3 independent experiments. (**C**) Quantification of LC3-II / LC3-I ratio of (**B**) by densitometry analysis. (**D**) Flow cytometry ratio analysis of *Mcl1^-/-^* and *Mcl1^-/-^Atg14*^CRISPR^ MEFs expressing mtKeima, treated with DFP [100µM] for 24h. Data are mean ± SEM for 4 independent experiments. (**E**) Immunoblot of *Mcl1^-/-^ and Mcl1^-/-^Fip200*^CRISPR^ MEFs, confirming loss of FIP200 protein. (**F**) Immunoblot of *Mcl1^-/-^ and Mcl1^-/-^Fip200*^CRISPR^ MEFs after serum starvation, with or without bafilomycin A1 treatment. Representative of 3 independent experiments. (**G**) Quantification of LC3-II / LC3-I ratio of (**F**) by densitometry analysis. (**H**) Flow cytometry ratio analysis of *Mcl1^-/-^* and *Mcl1^-/-^Fip200*^CRISPR^ MEFs expressing mtKeima, treated with DFP [100µM] for 24h. Data are mean ± SEM for 2 independent experiments. (**I**) Representative flow-cytometry histograms plotting the ratio between ex:560nm (pH4) and ex:488nm (pH7) at 24h, for both *Mcl1^-/-^* and *Mcl1^-/-^Atg14*^CRISPR^ MEF lines. (**J**) Flow cytometry ratio analysis of *Mcl1^-/-^* and *Mcl1^-/-^Atg14*^CRISPR^ MEFs expressing mtKeima, treated with DMSO, ABT-737 [1µM] ± QVD-OPh [20µM] for 8h or 24h*.* (**K**) Representative flow-cytometry histograms plotting the ratio between ex:560nm (pH4) and ex:488nm (pH7) at 24h, for both *Mcl1^-/-^* and *Mcl1^-/-^Fip200*^CRISPR^ MEF lines. (**L**) Flow cytometry ratio analysis of *Mcl1^-/-^* and *Mcl1^-/-^Fip200*^CRISPR^ MEFs expressing mtKeima, treated with DMSO, ABT-737 [1µM] ± QVD-OPh [20µM] for 8h or 24h*.*

**Figure S4 Knockout assessment and functional testing of HeLas lacking ATG3, ATG5, and ATG7.** (**A**) Immunoblot of *WT and ATG3 KO* HeLas, confirming loss of ATG3 protein. (**B**) Immunoblot of *WT and ATG3 KO* HeLas after serum starvation, with or without bafilomycin A1 treatment. Representative of 3 independent experiments. (**C**) Quantification of LC3-II / LC3-I ratio in (**B**) by densitometry analysis. (**D**) Immunoblot of *WT, ATG5 KO and ATG7 KO* HeLas, confirming loss of ATG5 and ATG7 protein. (**E**) Immunoblot of *WT, ATG5 KO and ATG7 KO* HeLas after serum starvation, with or without bafilomycin A1 treatment. Representative of 3 independent experiments. (**F**) Quantification of LC3-II / LC3-I ratio of (**E**) by densitometry analysis. (**G**) Flow cytometry ratio analysis of *WT, ATG3 KO, ATG5 KO and ATG7 KO* HeLas expressing mtKeima, treated with DFP [100µM] for 24h. Data are mean ± SEM for 3 independent experiments. (**H**) Representative flow-cytometry histograms plotting the ratio between ex:560nm (pH4) and ex:488nm (pH7) treated with DMSO, ABT-737 [1µM] ± QVD-OPh [20µM] for 24h, for both *WT, ATG5 KO, ATG3 KO, and ATG7 KO* HeLas lines. (**I-J**) Representative flow-cytometry histograms plotting the ratio between ex:560nm (pH4) and ex:488nm (pH7) of *Mcl1^-/-^* MEFs treated with DMSO, ABT-737 [1µM] ± QVD-OPh [20µM] in combination with (**I**) Wortmannin [1µM] for 4h, or (**J**) BafilomycinA1 [25nM] or Chloroquine [100µM] for 16h.

**Figure S5 Mitochondrial localisation of ubiquitin adaptor proteins P62, OPTN, and NDP52 after mitochondrial herniation.** (**A**) Schematic of mitochondrial morphology before and after mitochondrial herniation, with TFAM (yellow) moving from within TOMM20 (pink) to outside, and the recruitment of autophagy adaptor (blue) to yellow TFAM signal. (**B**) Recruitment of GFP-P62. Fluorescence intensity line-scan analysis of mitochondria from inset. (**C**) Recruitment of GFP-OPTN. Fluorescence intensity line-scan analysis of mitochondria from inset. (**D**) Recruitment of GFP-NDP52. Fluorescence intensity line-scan analysis of mitochondria from inset. (**E**) Flow cytometry ratio analysis of *Mcl1^-/-^* MEFs expressing mtKeima, treated for 8h, or 24h with DMSO, ABT-737 [1µM] ± QVD-OPh [20µM] in combination with either BX795 [1µM] or MRT68921 [1µM].

**Figure S6 The herniated inner mitochondrial membrane is ubiquitinated, and acts as a site for formation of engulfing autophagosome.** (**A**) Airyscan confocal imaging of *Mcl1^-/-^* MEFs expressing TOMM20-Halo (stained with JF646) (pink) and TFAM-mScarlet (yellow) treated with ABT-737 [1µM] and QVD-OPh [20µM] as shown in **Figure 5A**. Fluorescence intensity line-scan analysis of mitochondria from inset. (**B**) *Mcl1^-/-^* MEFs were exposed to ABT-737 [1µM] and QVD-OPh [20µM] or Antimycin A [4µM] and Oligomycin [10µM] for 4h, and crude mitochondrial isolates from these cells were then assessed by SDS-PAGE **Figure5B** for key mitochondrial proteins. CBB = Coomassie brilliant blue. Purity of mitochondrial isolates was assessed by probing for B-ACTIN and VDAC in whole cell lysates and compared to crude mitochondrial isolates. (**C**) Airyscan confocal imaging of *Mcl1^-/-^* MEFs expressing TOMM20-Halo (stained with JF646) (pink) and GFP-OPTN (green) treated with ABT-737 [1µM] and QVD-OPh [20µM] for 4h, and probed for ubiquitin (light blue). Inset of herniating mitochondria shown on right. (**D**) Airyscan confocal imaging of *Mcl1^-/-^* MEFs expressing TOMM20-Halo (stained with JF646) (pink) and GFP-NDP52 (green) treated with ABT-737 [1µM] and QVD-OPh [20µM] for 4h, and probed for ubiquitin (light blue). Inset of herniating mitochondria shown on right. (**E**) Airyscan confocal imaging of *Mcl1^-/-^* MEFs expressing TOMM20-Halo (stained with JF646) (pink) and GFP-LC3B (green) treated with either Bafilomycin A1 [25nM] or Bafilomycin A1 [25nM], QVD-OPh [20µM] and ABT-737 [1µM], for 2h and stained for conjugated ubiquitin (light blue). (**F-G**) Airyscan confocal imaging of *Mcl1^-/-^* MEFs expressing TOMM20-Halo (stained with JF646) (pink) and GFP-LC3B (green) treated with ABT-737 [1µM] and QVD-OPh [20µM] with (**F**) or without (**G**) TAK243 [1µM] as shown in **Figure 6A**. Fluorescence intensity line-scan analysis of mitochondria from inset. (**H**) Inset and fluorescence intensity line-scan analysis of mitochondria from inset image in (**E**).

**Figure S7 Ubiquitin of inner mitochondrial membrane is essential for apoptotic mitophagy.** (**A**) Representative flow-cytometry histograms plotting the ratio between ex:560nm (pH4) and ex:488nm (pH7) for *Mcl1^-/-^* MEFs treated with DMSO and QVD-OPh [20µM] + ABT-737 [1µM], with increasing doses of TAK243 [500nM and 1µM] for 24h. (**B**) Immunoblot of *Mcl1^-/-^* MEFs treated with MG132. (**C**) Representative flow-cytometry histograms plotting the ratio between ex:560nm (pH4) and ex:488nm (pH7) for *Mcl1^-/-^* MEFs treated with DMSO and QVD-OPh [20µM] + ABT-737 [1µM], with or without MG132 for 4h. (**D**) Immunoblot of WT vs Penta KO HeLas, confirming loss of protein of TAX1BP1, NDP52, OPTN, NBR1, and P62. (**E-F**) Flow cytometry ratio analysis of WT and Penta KO HeLas expressing BFP-Parkin and mtKeima, 2h with DMSO or Antimycin [4µM] and Oligomycin [10µM]. (**E**) Graph represents median ratio after treatment, normalised to DMSO control. (**F**) Representative flow-cytometry histograms plotting the ratio between ex:560nm (pH4) and ex:488nm (pH7). (**G**) WT HeLas and Penta KO HeLas treated with ABT-737 [2µM] and S63845 [5µM] for 4h. Fixed and stained with anti-TOMM20 (magenta) & anti-DNA (green) imaged on LSM980 Confocal.
