# Supplementary figures and images for: Exposure of the inner mitochondrial membrane triggers apoptotic mitophagy
Source: Cell Death Differ. 2024 Feb 23;31(3):335–47. doi: 10.1038/s41418-024-01260-2 (PMC10923902; doi:10.1038/s41418-024-01260-2)

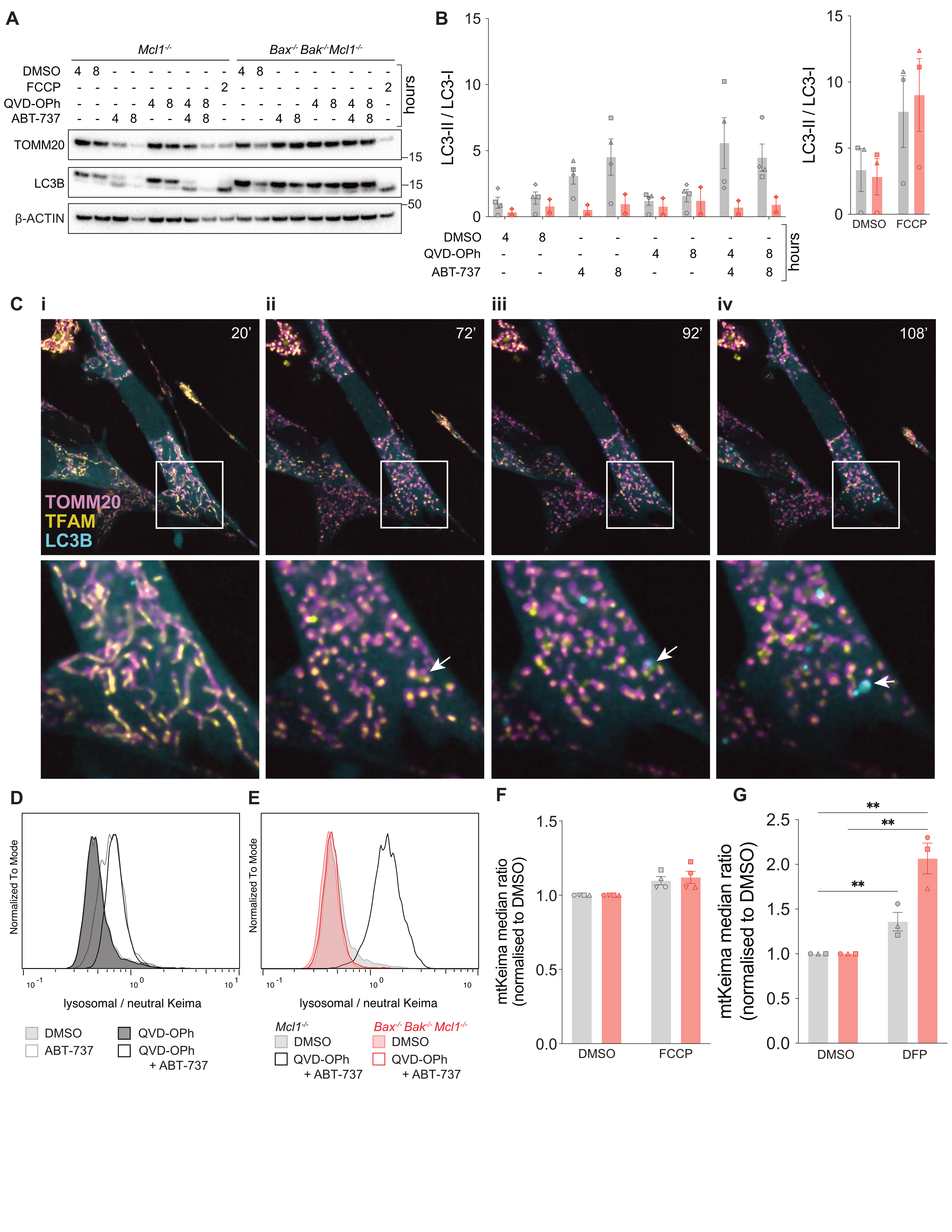

Supplement: Supplementary file 2 — Figure S1 [file 41418_2024_1260_MOESM2_ESM.png]

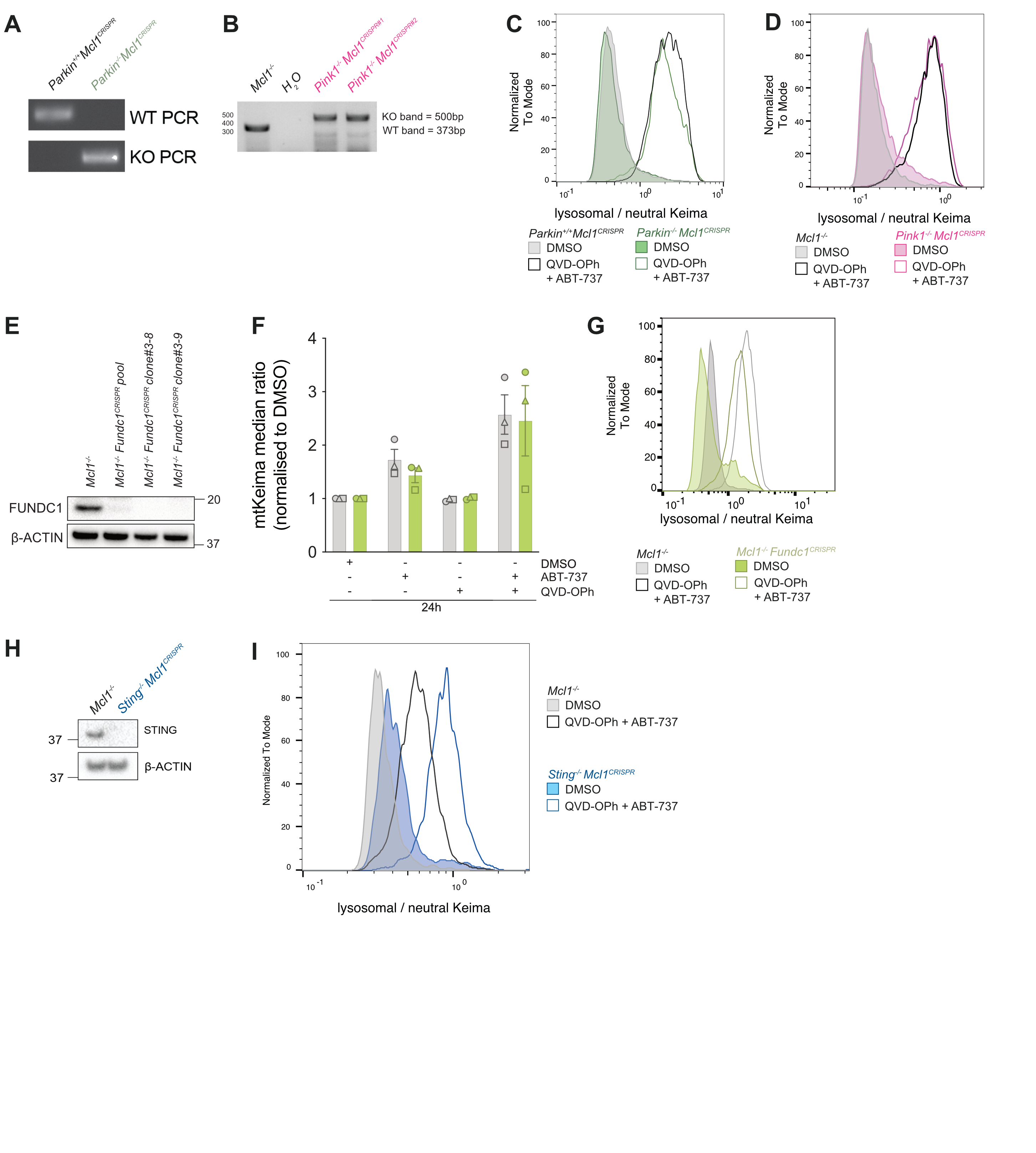

Supplement: Supplementary file 3 — Figure S2 [file 41418_2024_1260_MOESM3_ESM.png]

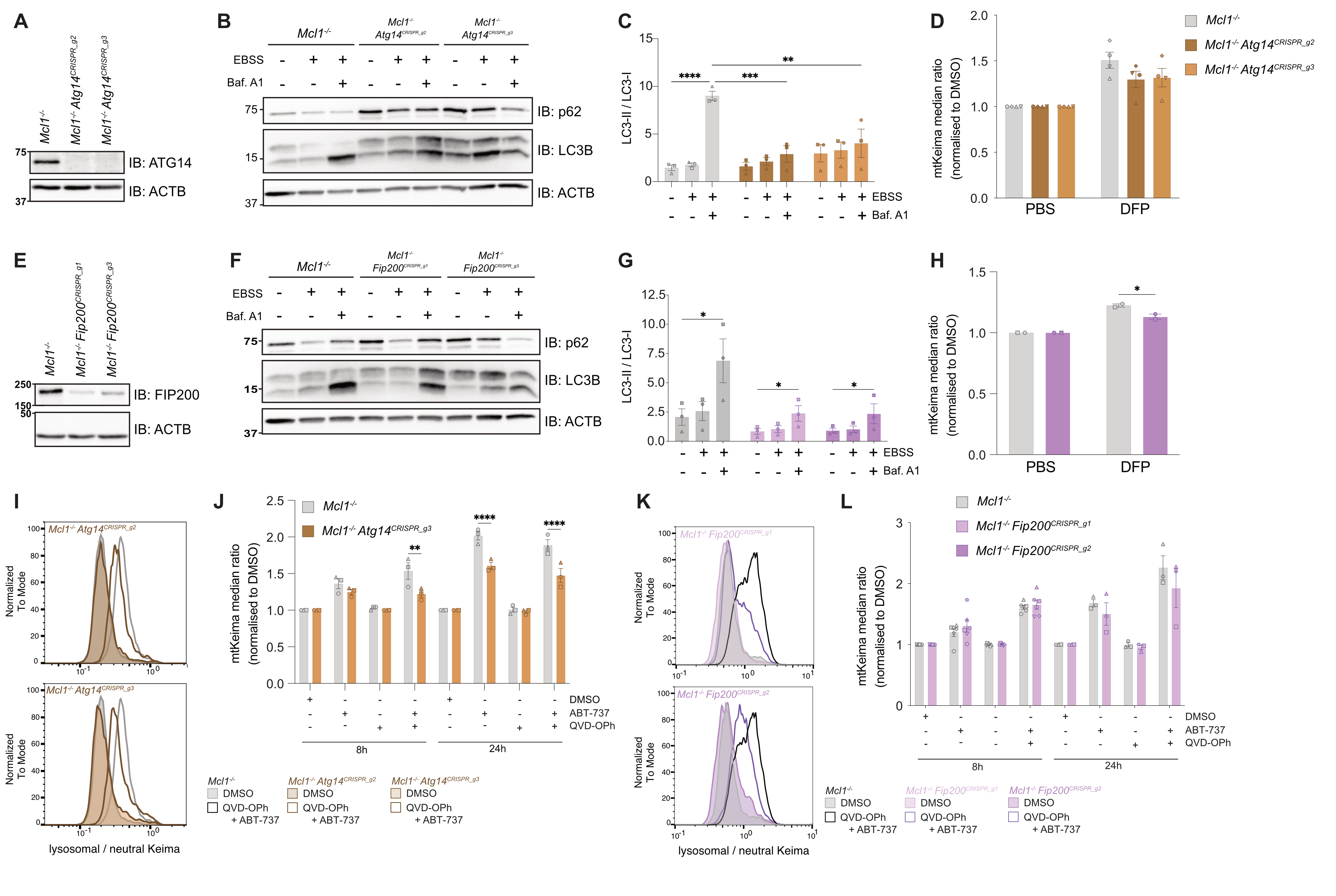

Supplement: Supplementary file 4 — Figure S3 [file 41418_2024_1260_MOESM4_ESM.png]

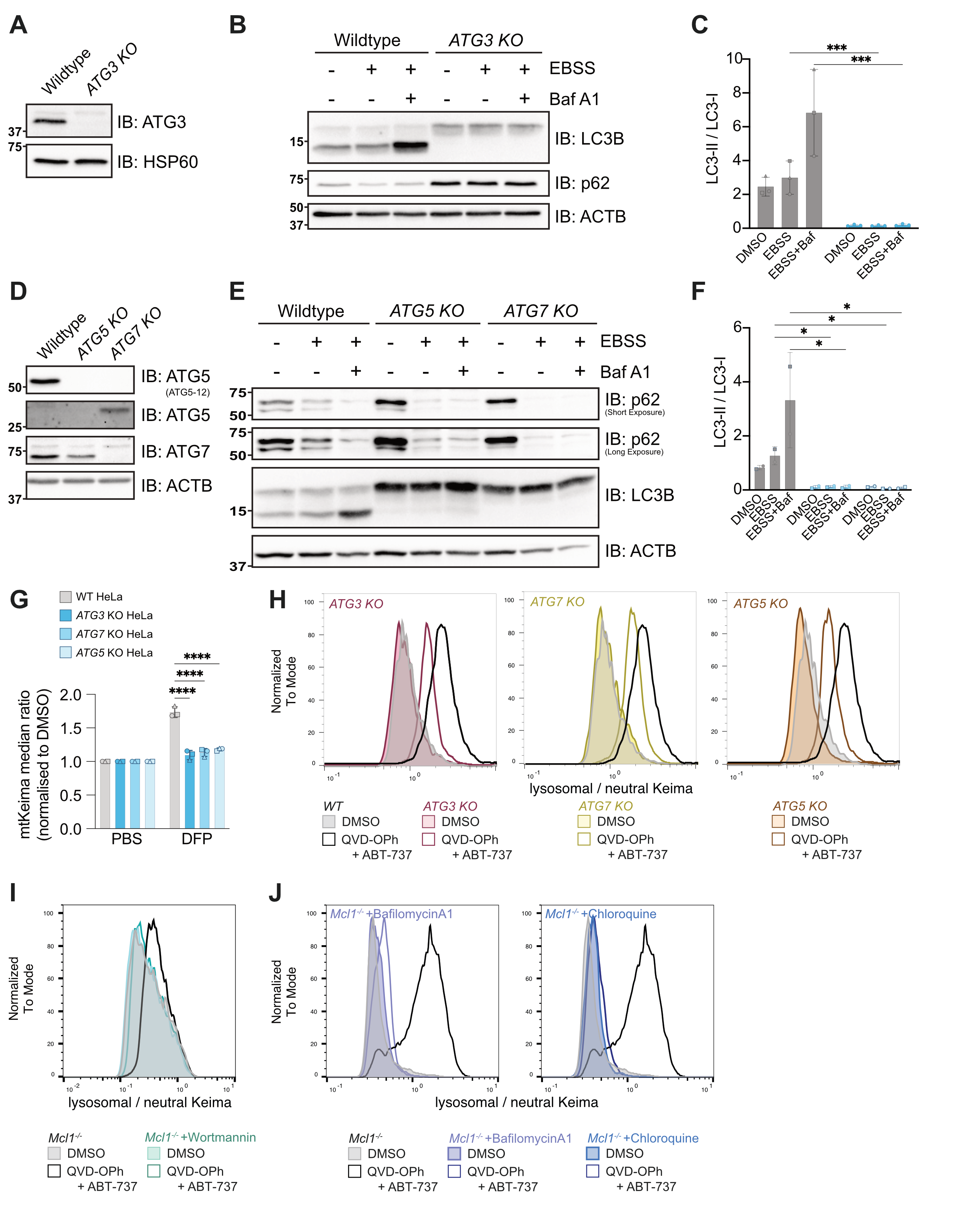

Supplement: Supplementary file 5 — Figure S4 [file 41418_2024_1260_MOESM5_ESM.png]

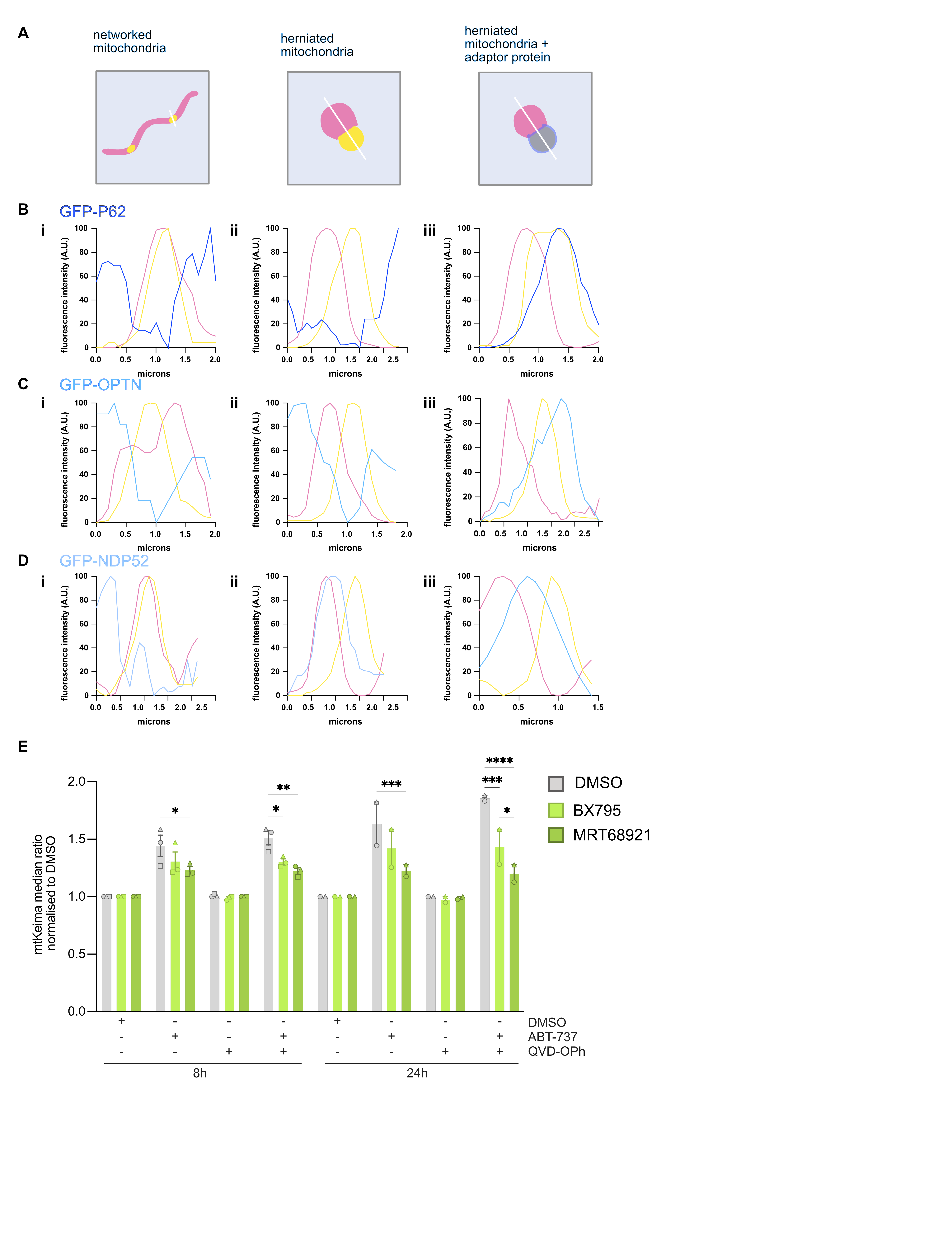

Supplement: Supplementary file 6 — Figure S5 [file 41418_2024_1260_MOESM6_ESM.png]

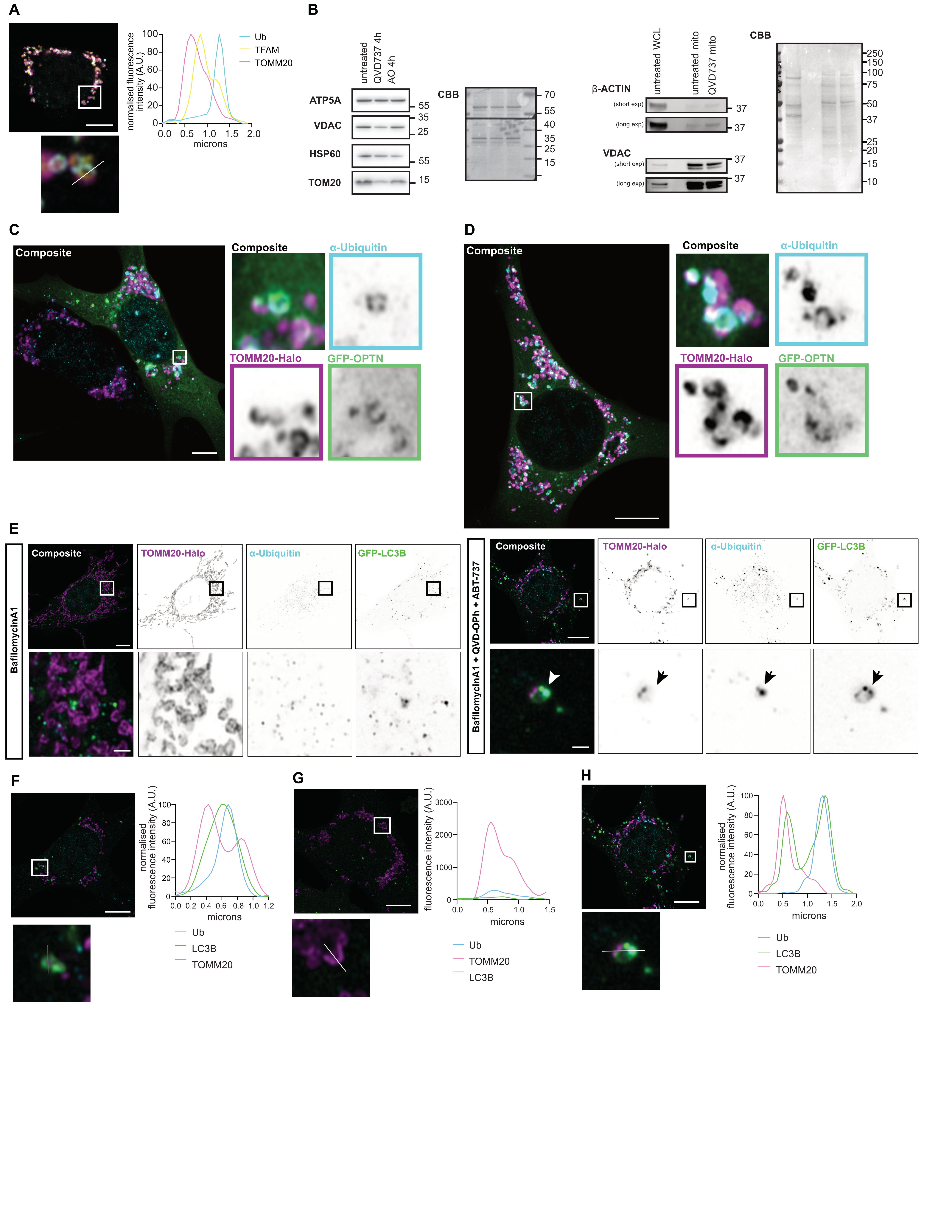

Supplement: Supplementary file 7 — Figure S6 [file 41418_2024_1260_MOESM7_ESM.png]

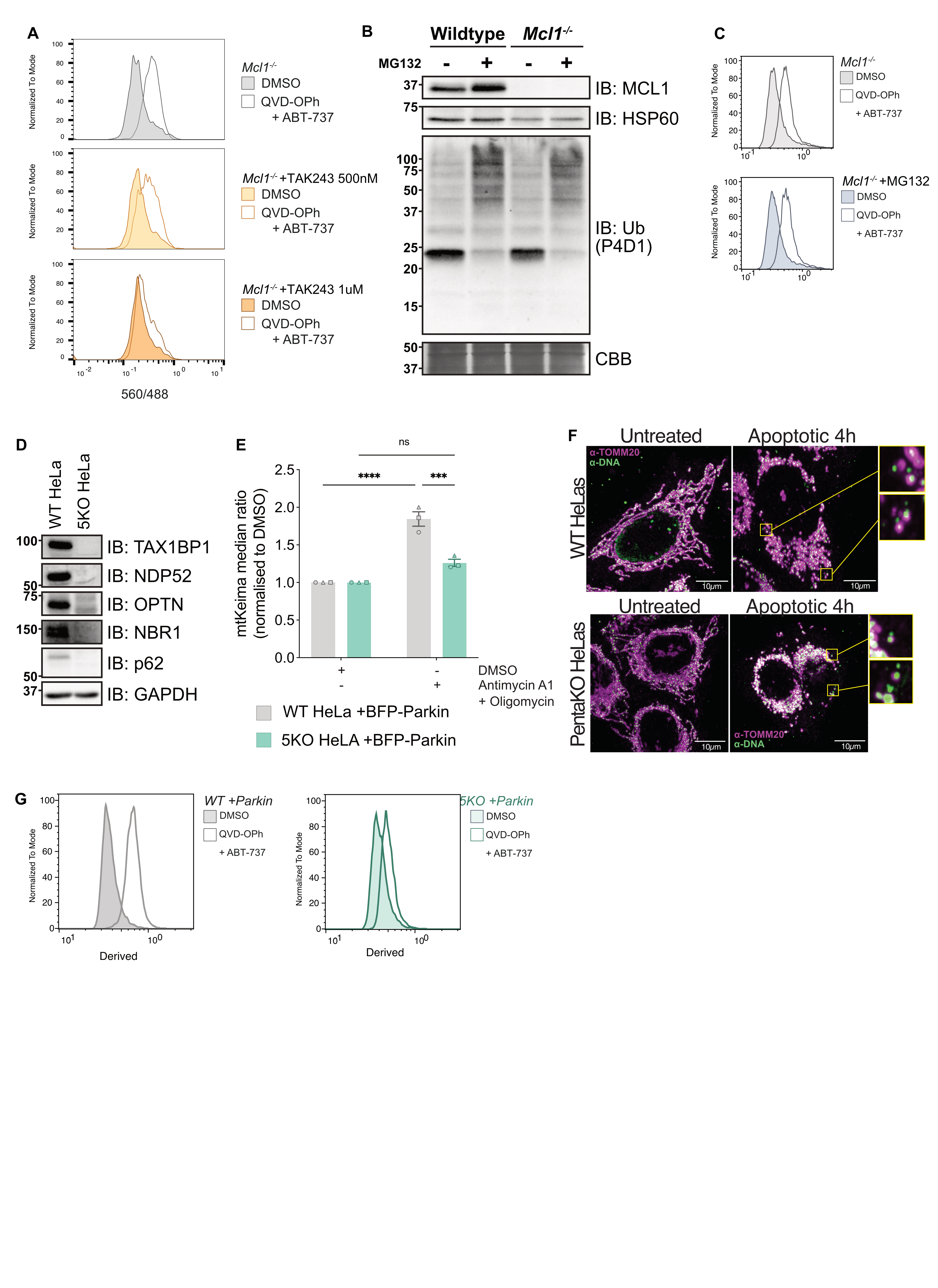

Supplement: Supplementary file 8 — Figure S7 [file 41418_2024_1260_MOESM8_ESM.png]
