# Supplementary material for: Exposure of the inner mitochondrial membrane triggers apoptotic mitophagy
Source: Cell Death Differ. 2024 Feb 23;31(3):335–47. doi: 10.1038/s41418-024-01260-2 (PMC10923902; doi:10.1038/s41418-024-01260-2)
Supplement: Supplementary file 14 — WB Supplementary Figures [file 41418_2024_1260_MOESM14_ESM.pdf]

Figure 1B

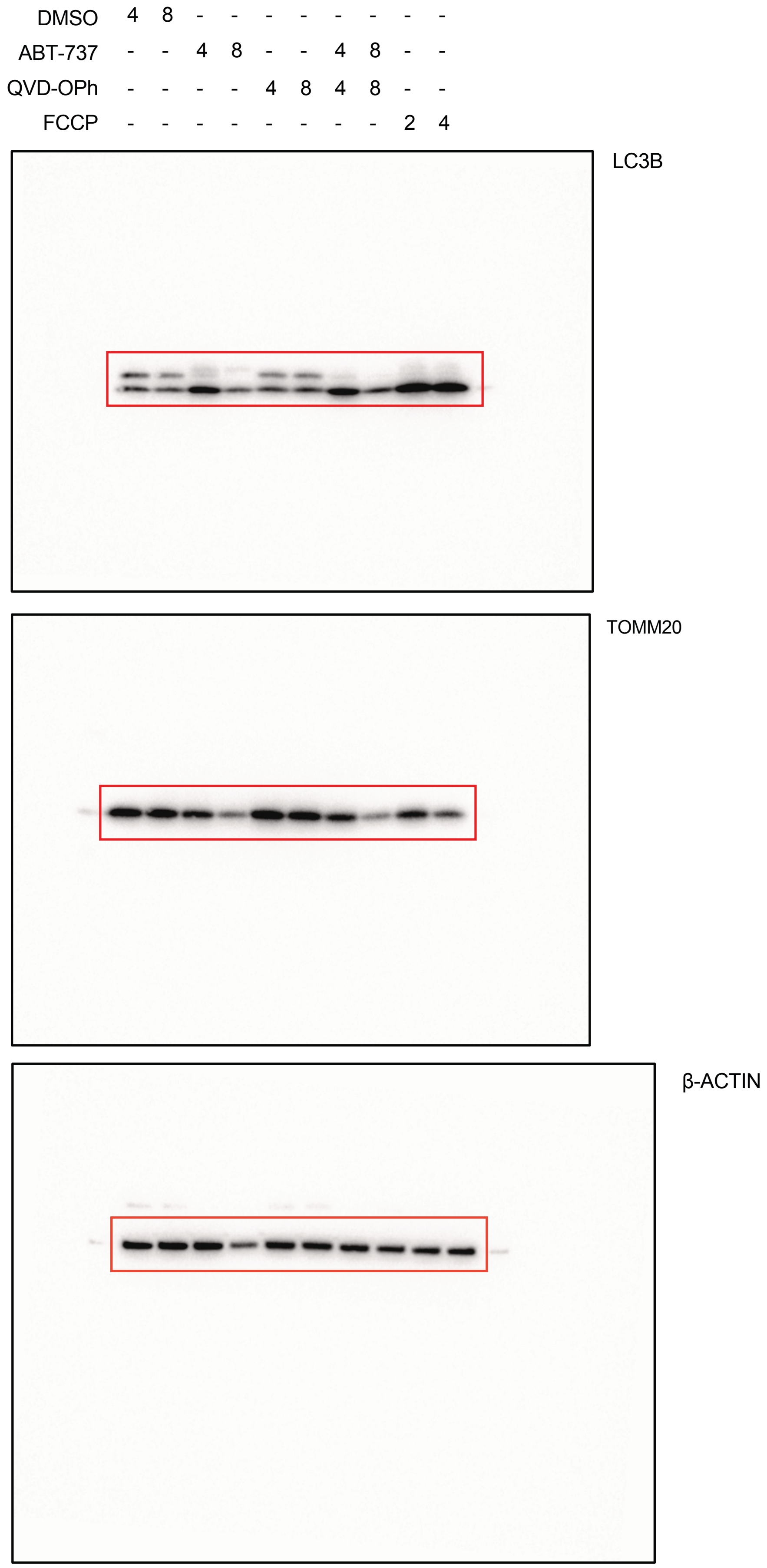

Figure 5B

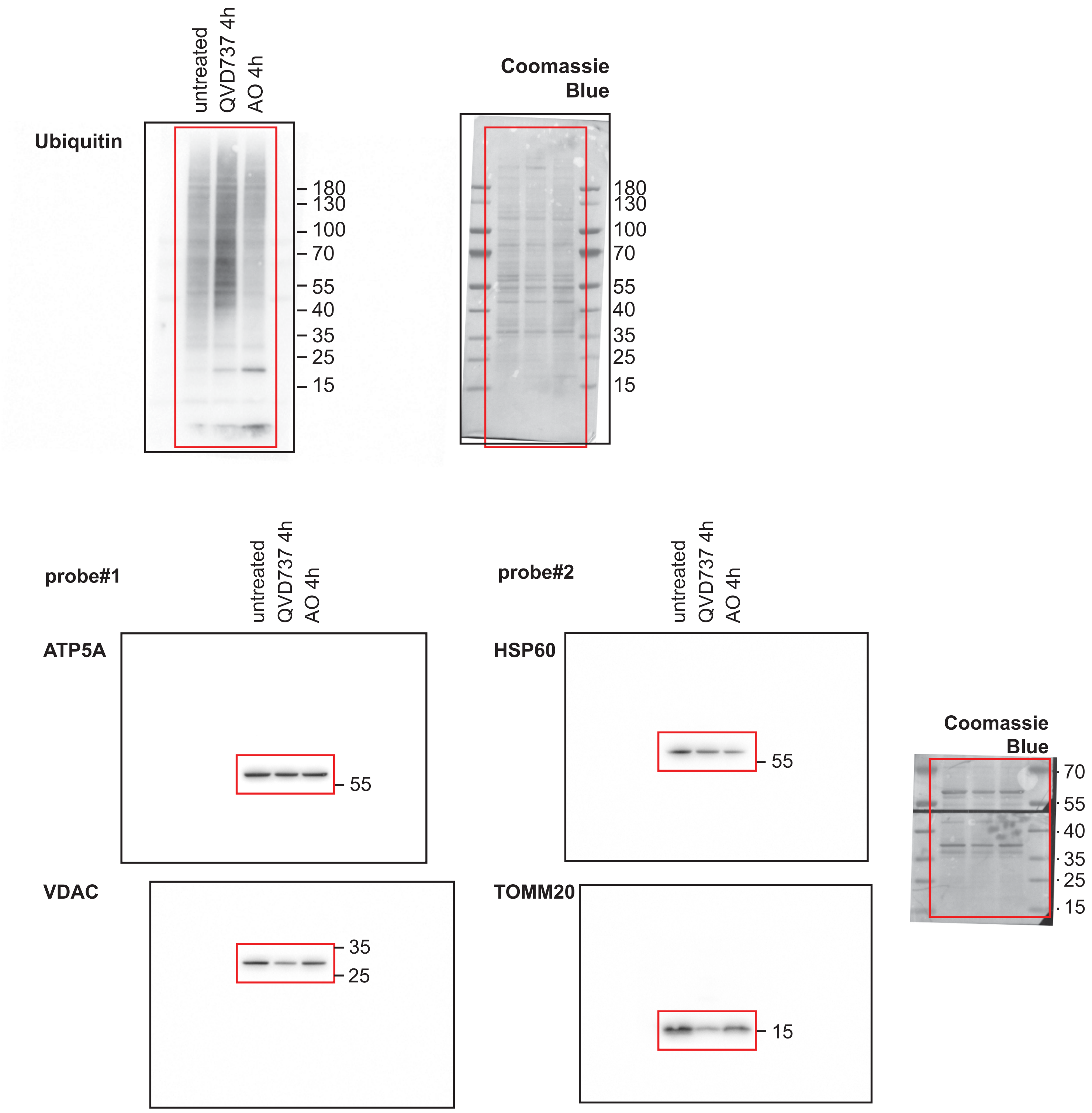

untreated
QVD737 4h
AO 4h
Ubiquitin
180
130
100
70
55
40
35
25
15
Coomassie Blue
probe#1
probe#2
ATP5A
VDAC
HSP60
TOMM20

Figure S1A

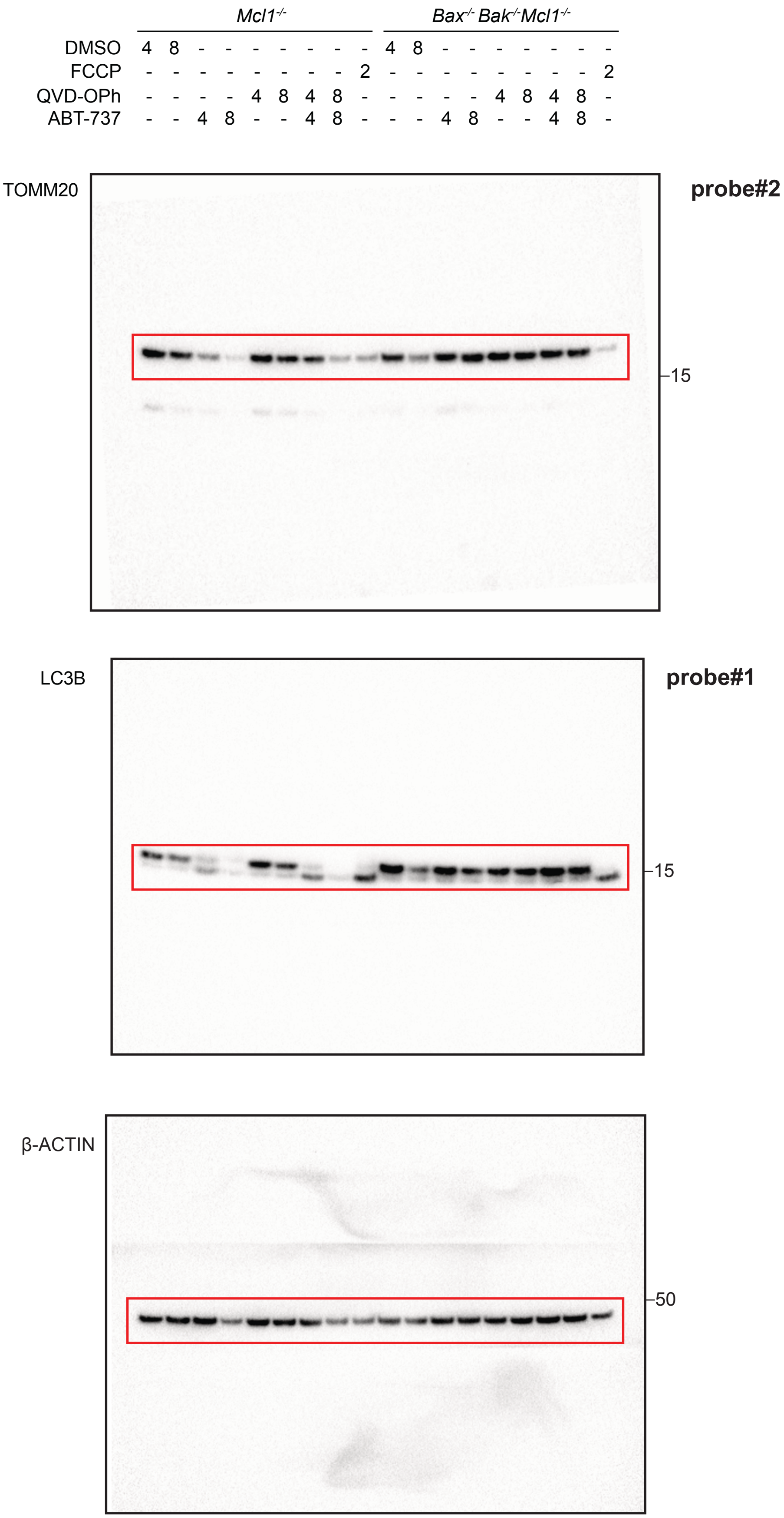

Mcl1-/-
Bax-/- Bak-/-Mcl1-/-
DMSO 4 8 - - - - - - - 4 8 - - - - - - -
FCCP - - - - - - - - 2 - - - - - - - - 2
QVD-OPh - - - - 4 8 4 8 - - - - - 4 8 4 8 -
ABT-737 - - 4 8 - - 4 8 - - - 4 8 - - 4 8 -
TOMM20
probe#2
15
LC3B
probe#1
15
β-ACTIN
50

Figure S2H

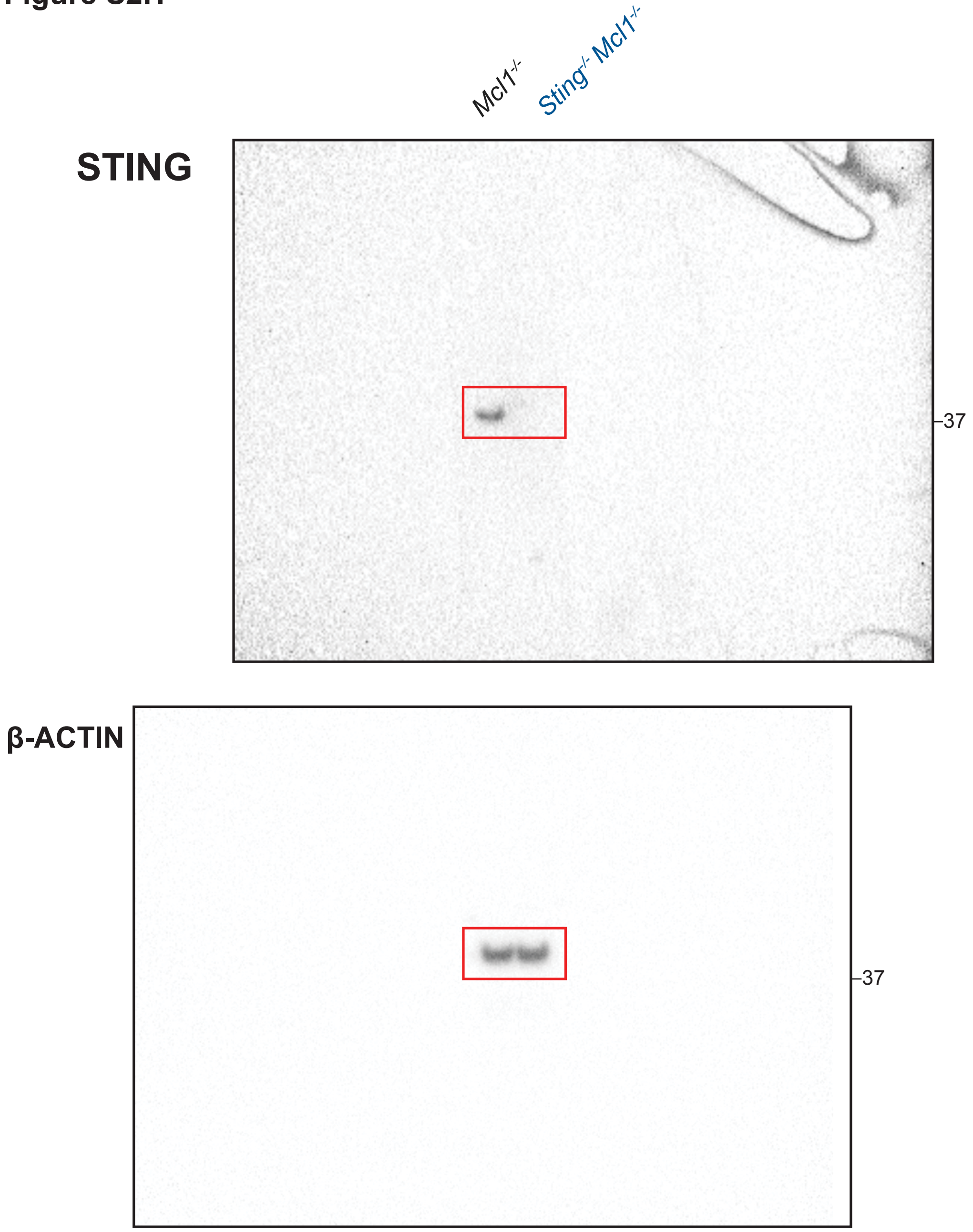

Figure S3A

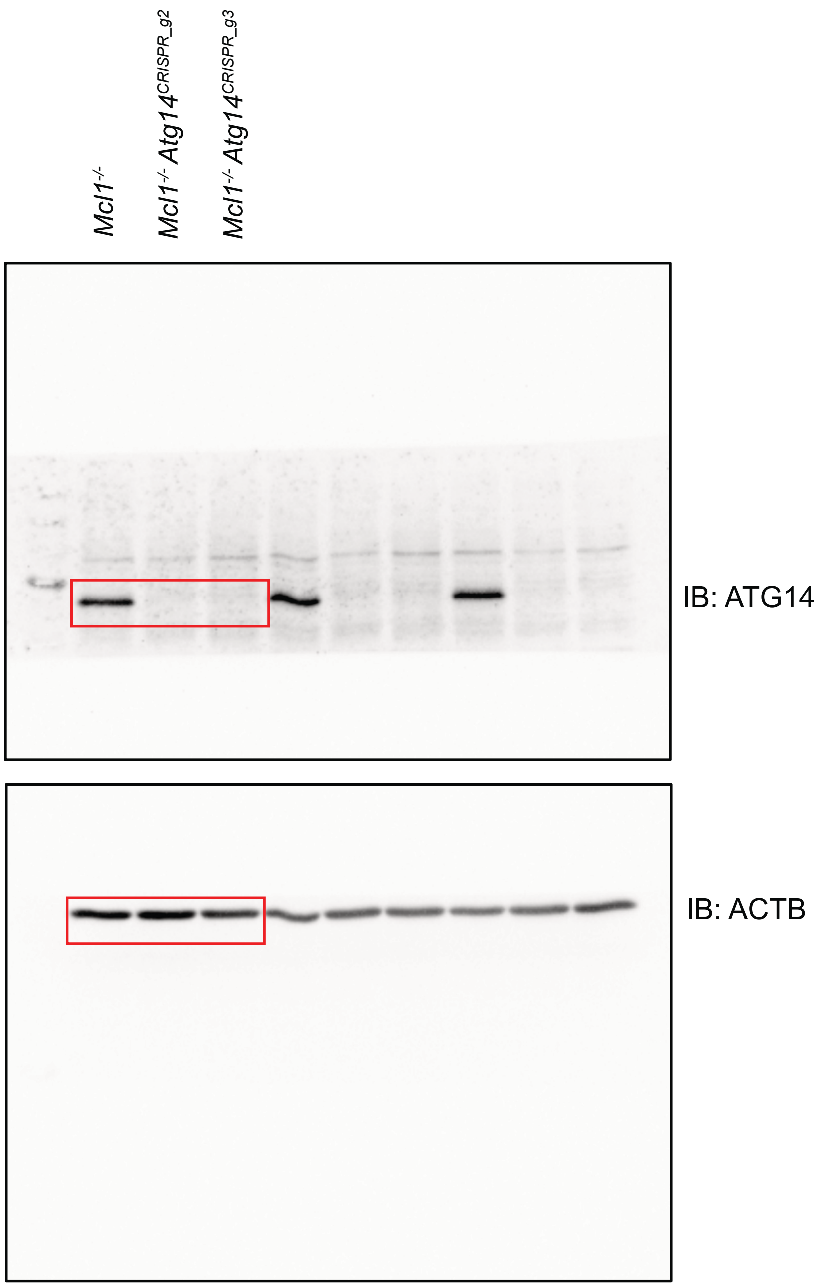

$Mcl1^{-/-}$
$Mcl1^{-/-}$ $Atg14^{CRISPR\_g2}$
$Mcl1^{-/-}$ $Atg14^{CRISPR\_g3}$
IB: ATG14
IB: ACTB

Figure S3B

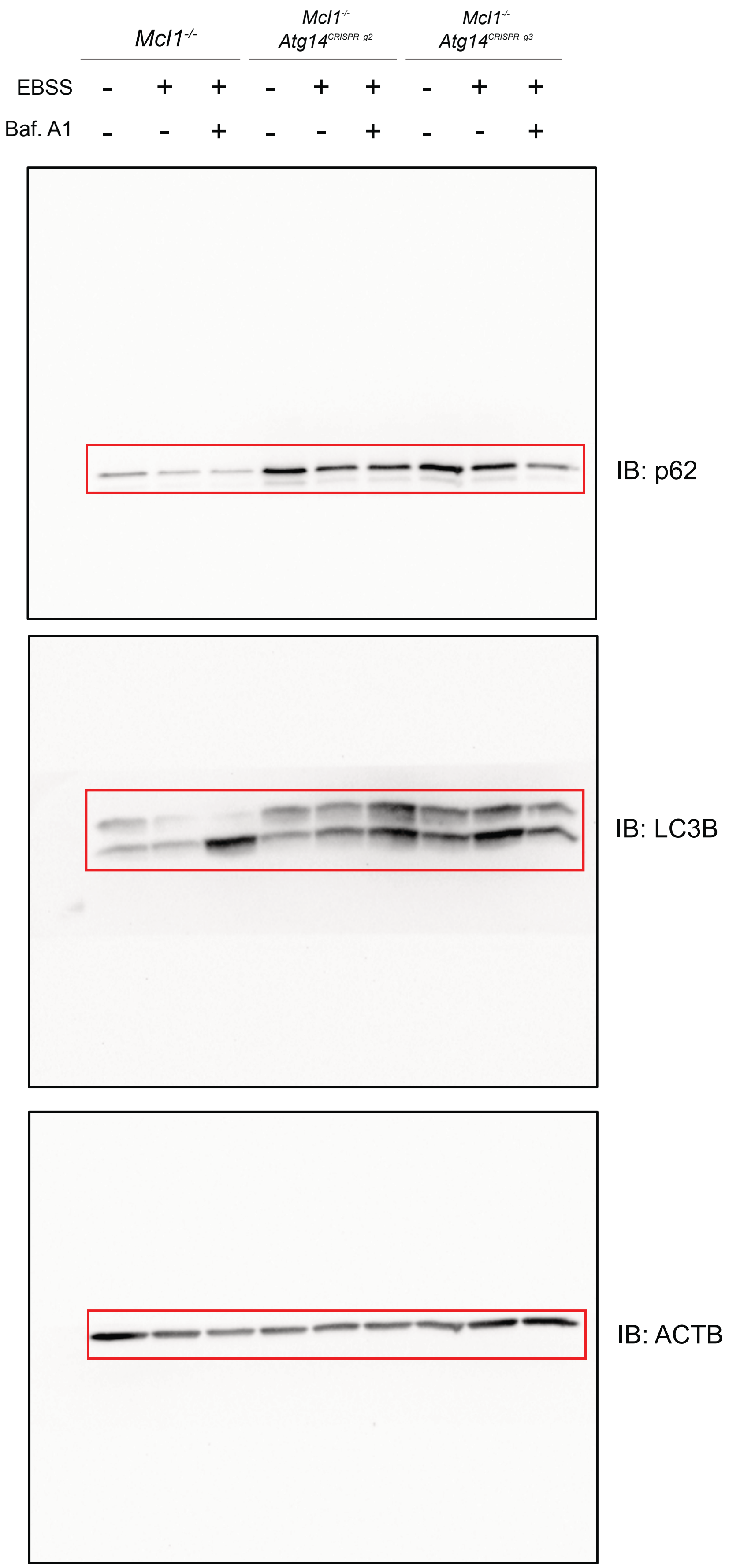

Mcl1-/-
Mcl1-/- Atg14CRISPR_g2
Mcl1-/- Atg14CRISPR_g3
EBSS - + + - + + - + +
Baf. A1 - - + - - + - - +
IB: p62
IB: LC3B
IB: ACTB

Figure S3E

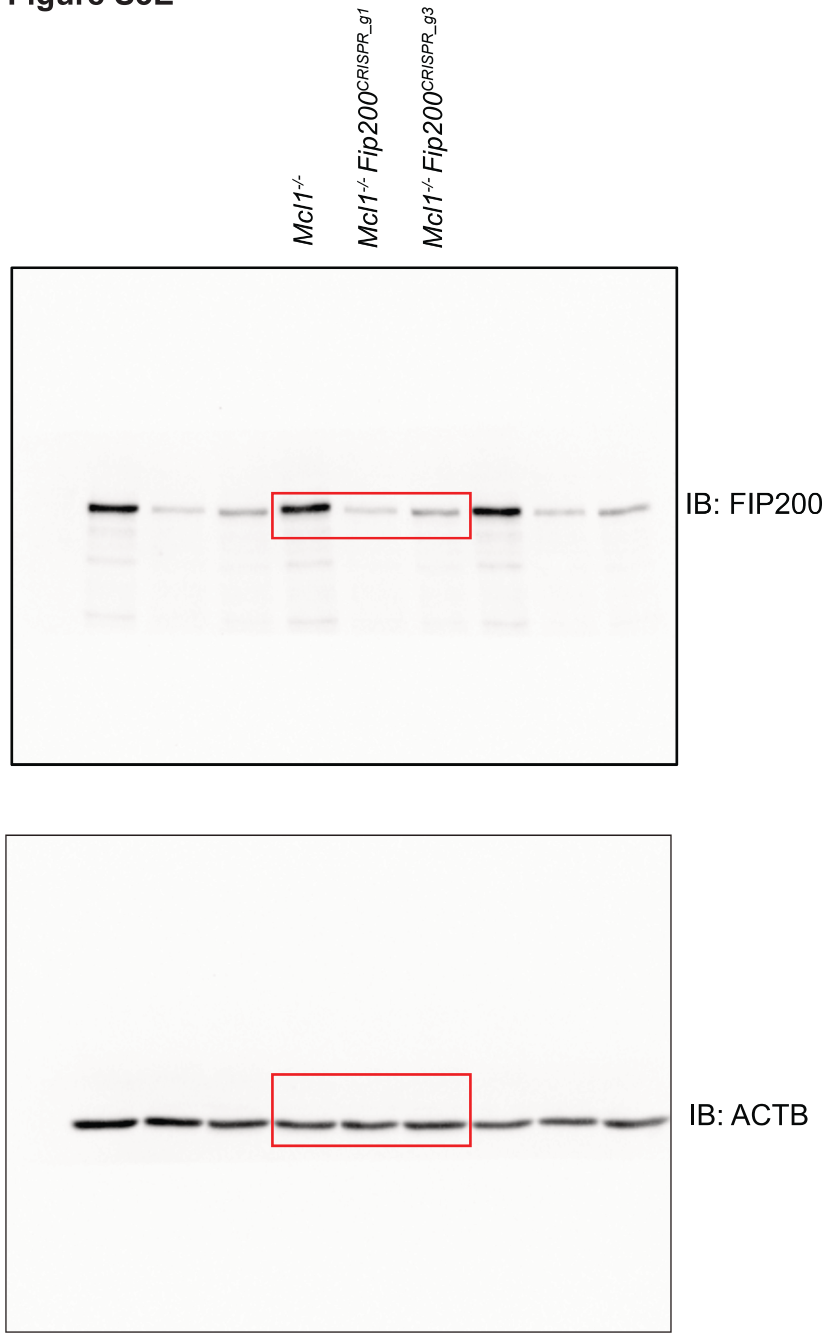
Mcl1-/-
Mcl1-/- Fip200CRISPR_g1
Mcl1-/- Fip200CRISPR_g3
IB: FIP200
IB: ACTB

Figure S3F

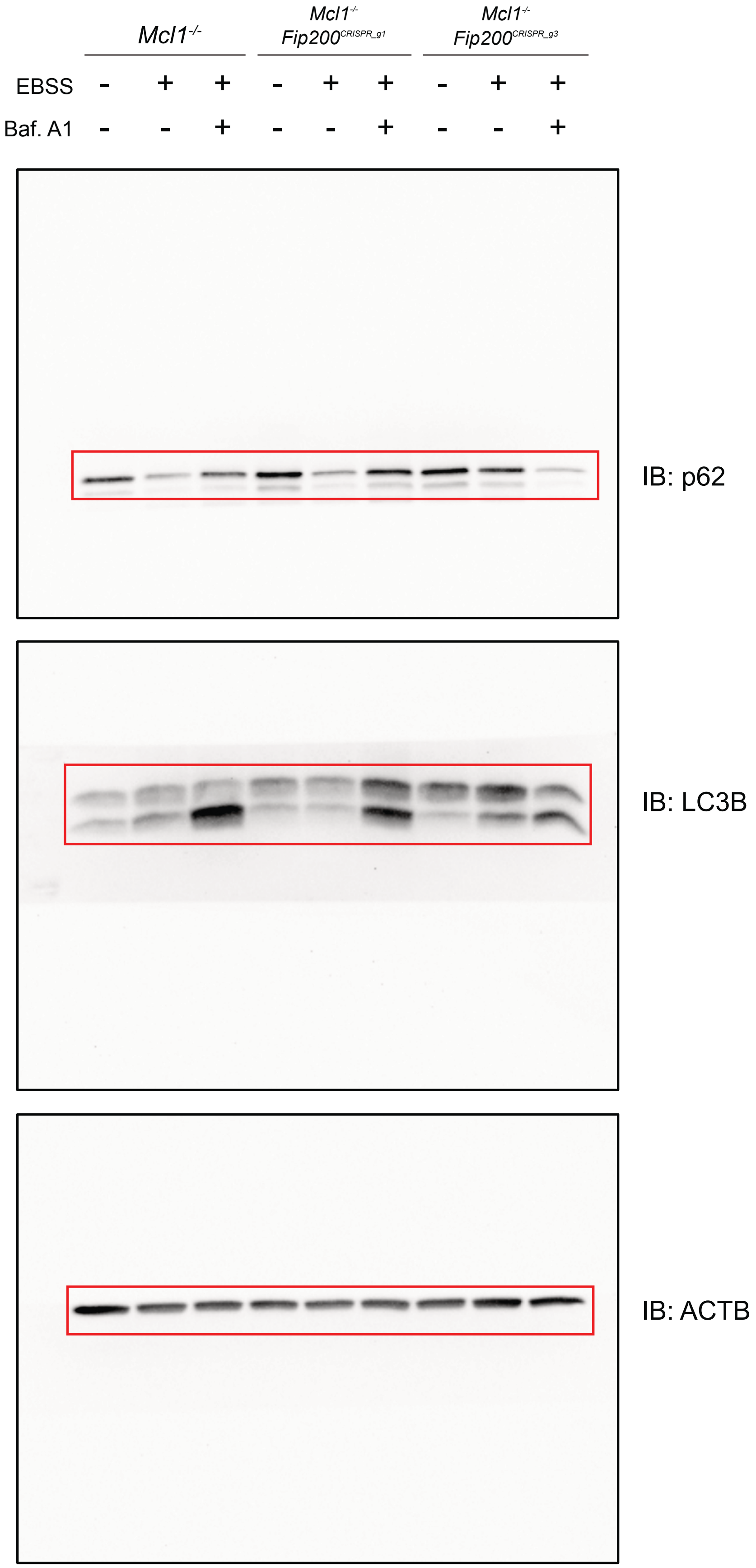
Mcl1-/-
Mcl1-/- Fip200CRISPR_g1
Mcl1-/- Fip200CRISPR_g3
EBSS - + + - + + - + +
Baf. A1 - - + - - + - - +
IB: p62
IB: LC3B
IB: ACTB

Figure S4A

Wildtype
$ATG3^{-/-}$

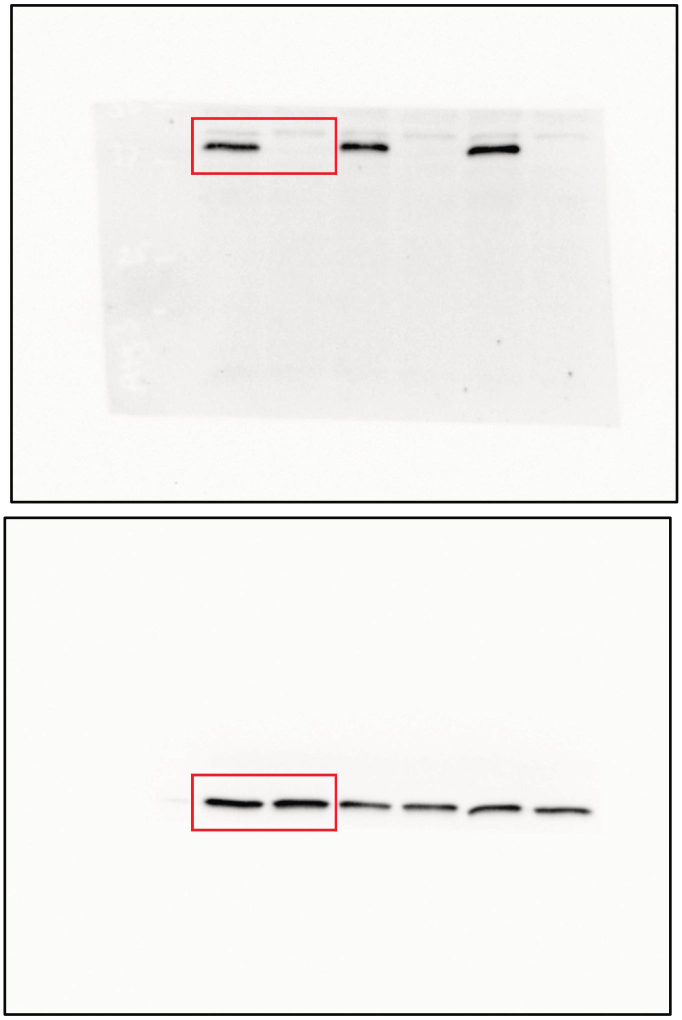

IB: ATG3

IB: HSP60

Figure S4B

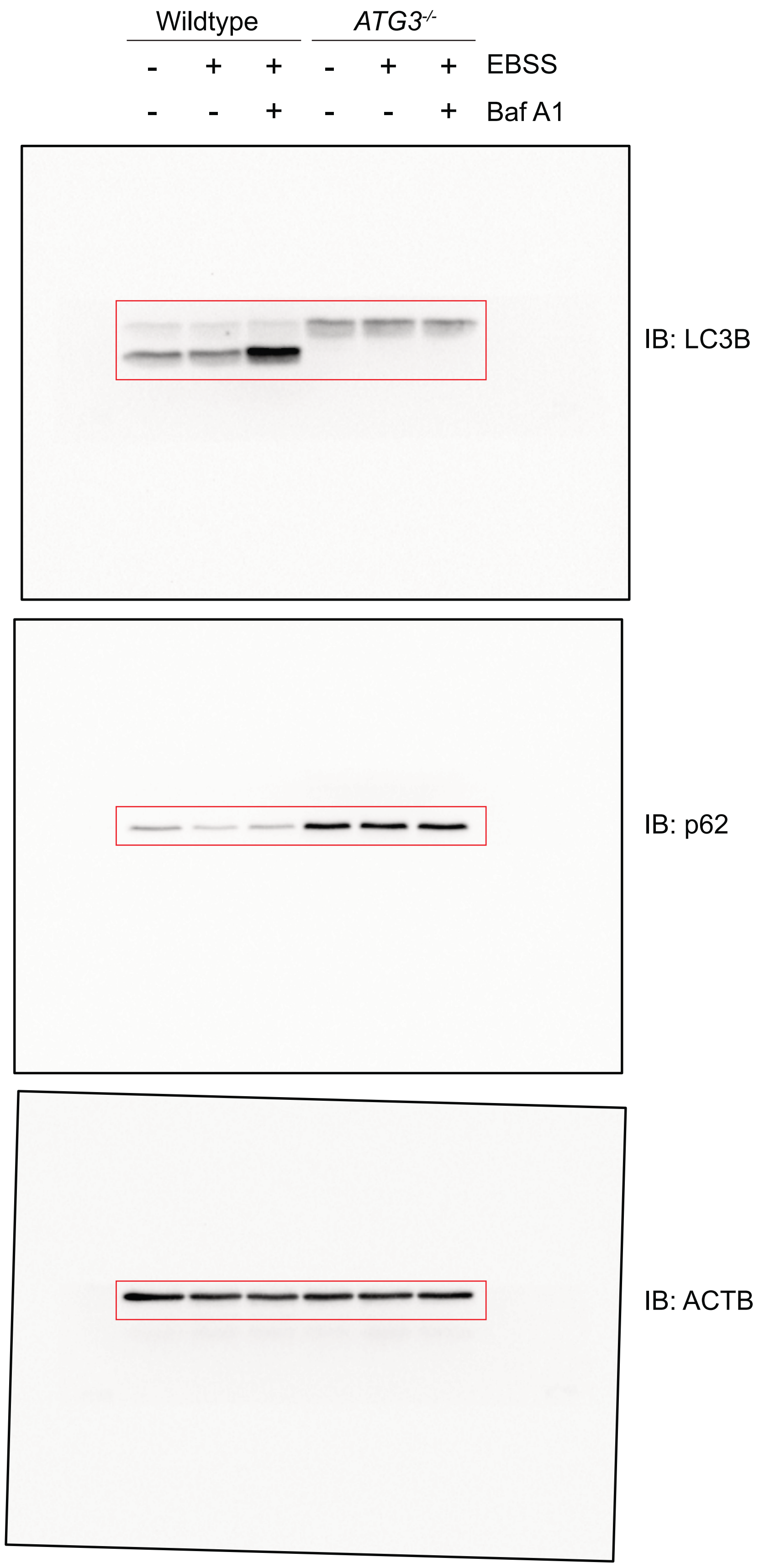

Figure S4D

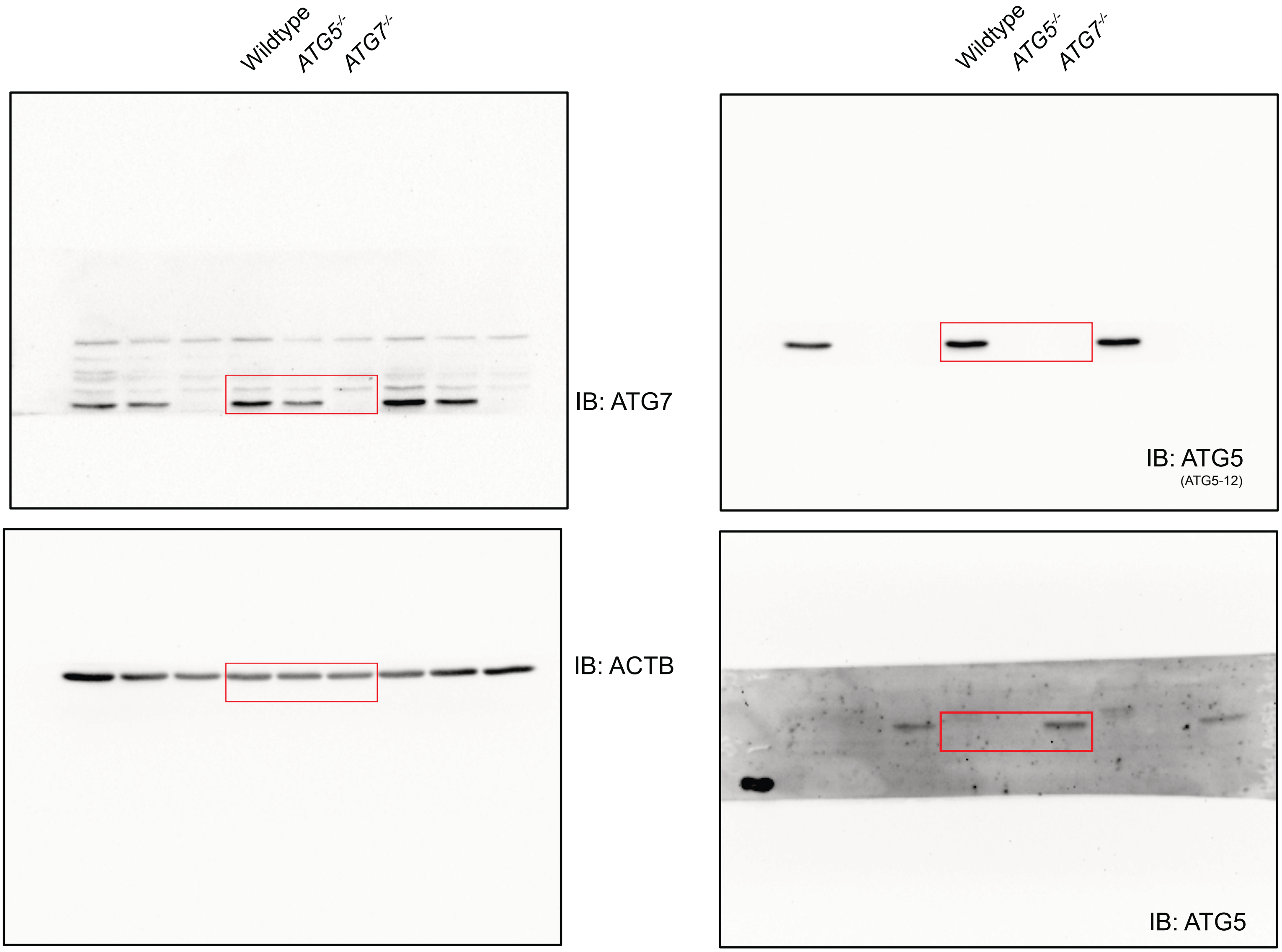
Wildtype
ATG5-/-
ATG7-/-
IB: ATG7
Wildtype
ATG5-/-
ATG7-/-
IB: ATG5
(ATG5-12)
IB: ACTB
IB: ATG5

Figure S4E

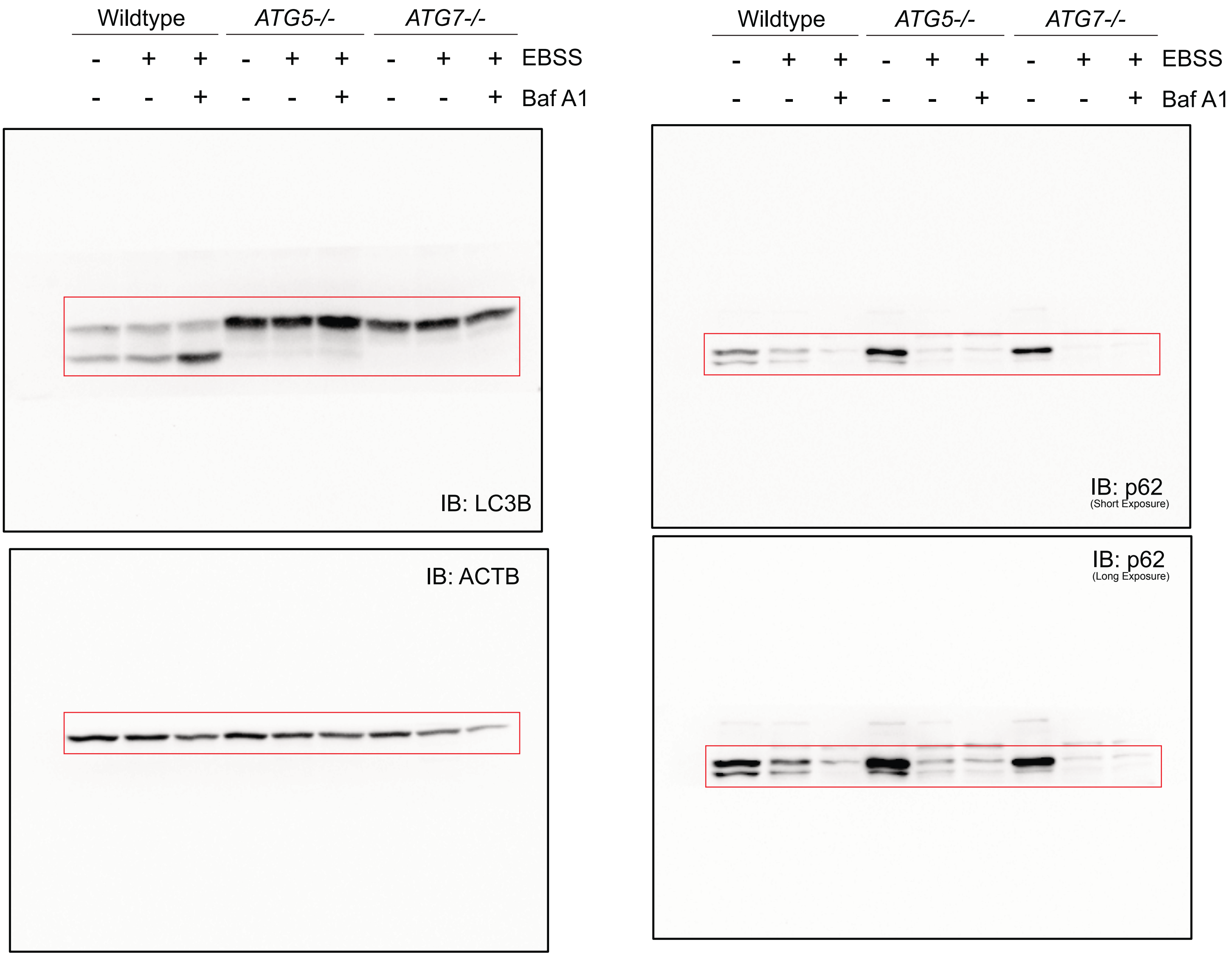
Wildtype
ATG5-/-
ATG7-/-
- + + - + + - + + EBSS
- - + - - + - - + Baf A1
IB: LC3B
IB: ACTB
Wildtype
ATG5-/-
ATG7-/-
- + + - + + - + + EBSS
- - + - - + - - + Baf A1
IB: p62
(Short Exposure)
IB: p62
(Long Exposure)

Figure S6B

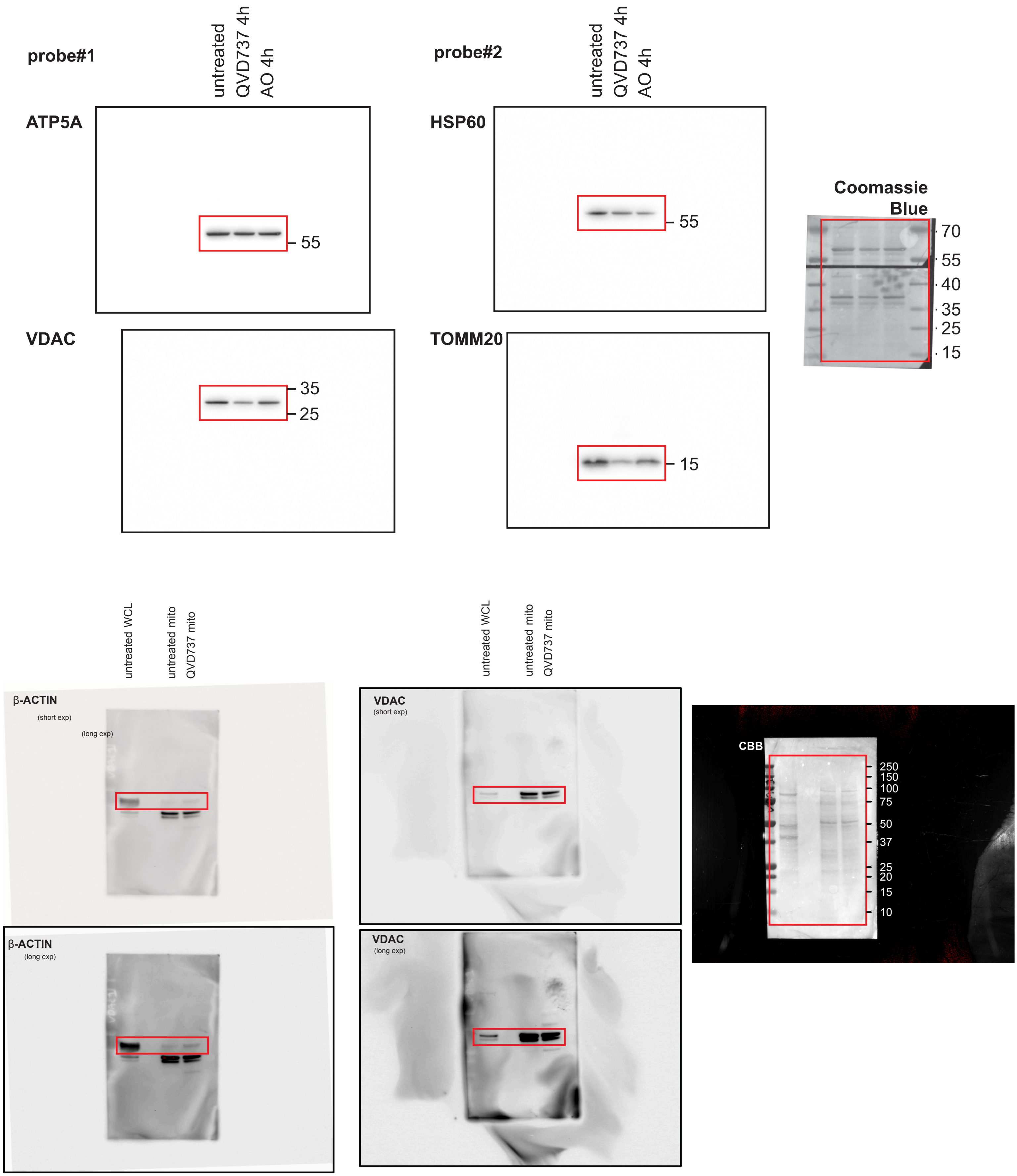

probe#1
untreated
QVD737 4h
AO 4h
ATP5A
55
VDAC
35
25
probe#2
untreated
QVD737 4h
AO 4h
HSP60
55
TOMM20
15
Coomassie Blue
70
55
40
35
25
15
untreated WCL
untreated mito
QVD737 mito
β-ACTIN
(short exp)
(long exp)
β-ACTIN
(long exp)
untreated WCL
untreated mito
QVD737 mito
VDAC
(short exp)
VDAC
(long exp)
CBB
250
150
100
75
50
37
25
20
15
10

Figure S7B

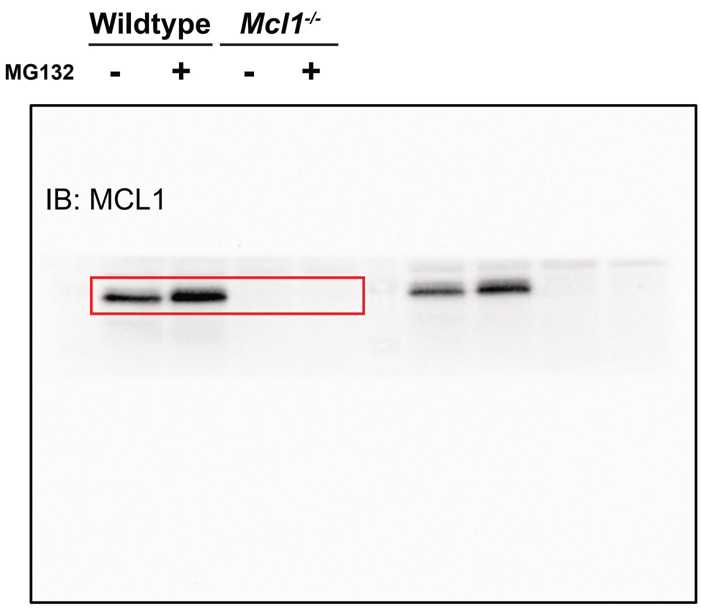
Wildtype
Mcl1-/-
MG132 - + - +
IB: MCL1

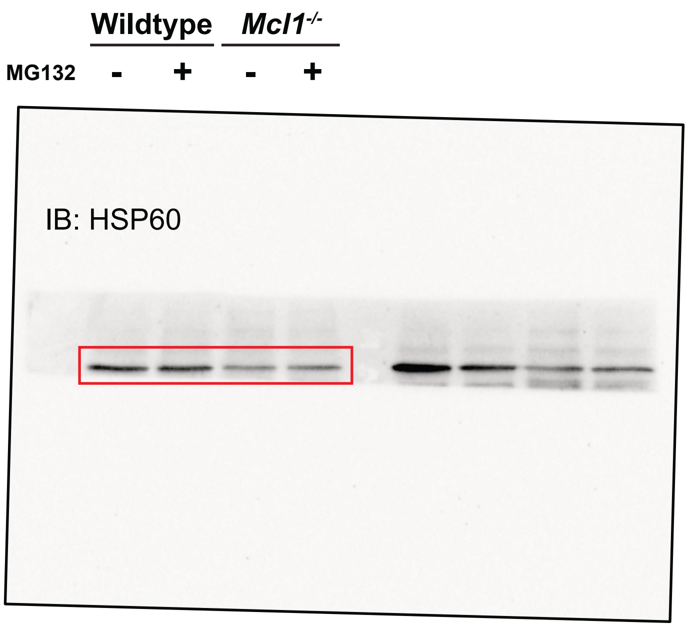
Wildtype
Mcl1-/-
MG132 - + - +
IB: HSP60

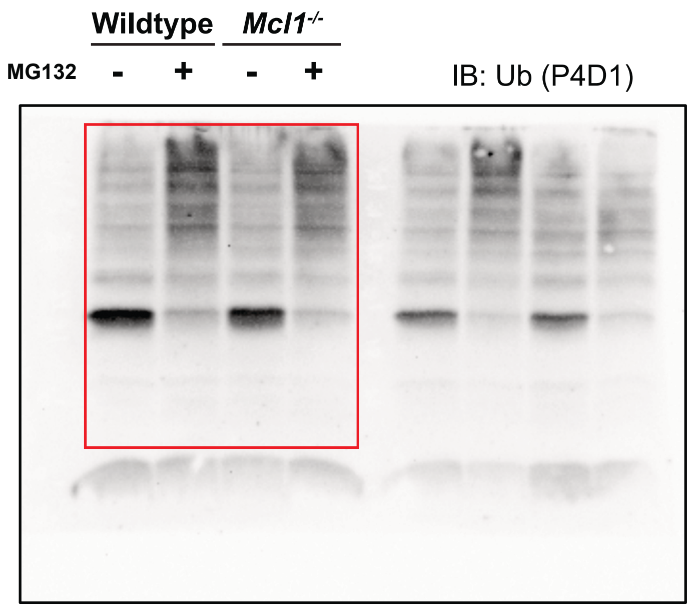
Wildtype
Mcl1-/-
MG132 - + - +
IB: Ub (P4D1)

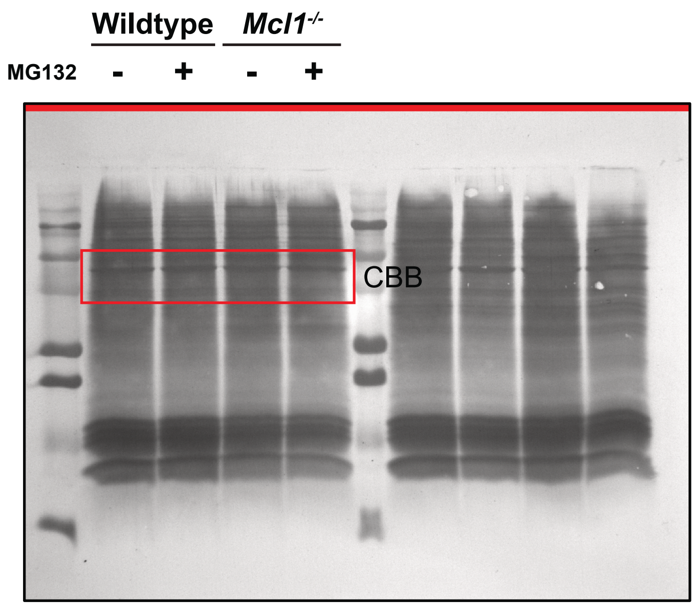
Wildtype
Mcl1-/-
MG132 - + - +
CBB

Figure S7D

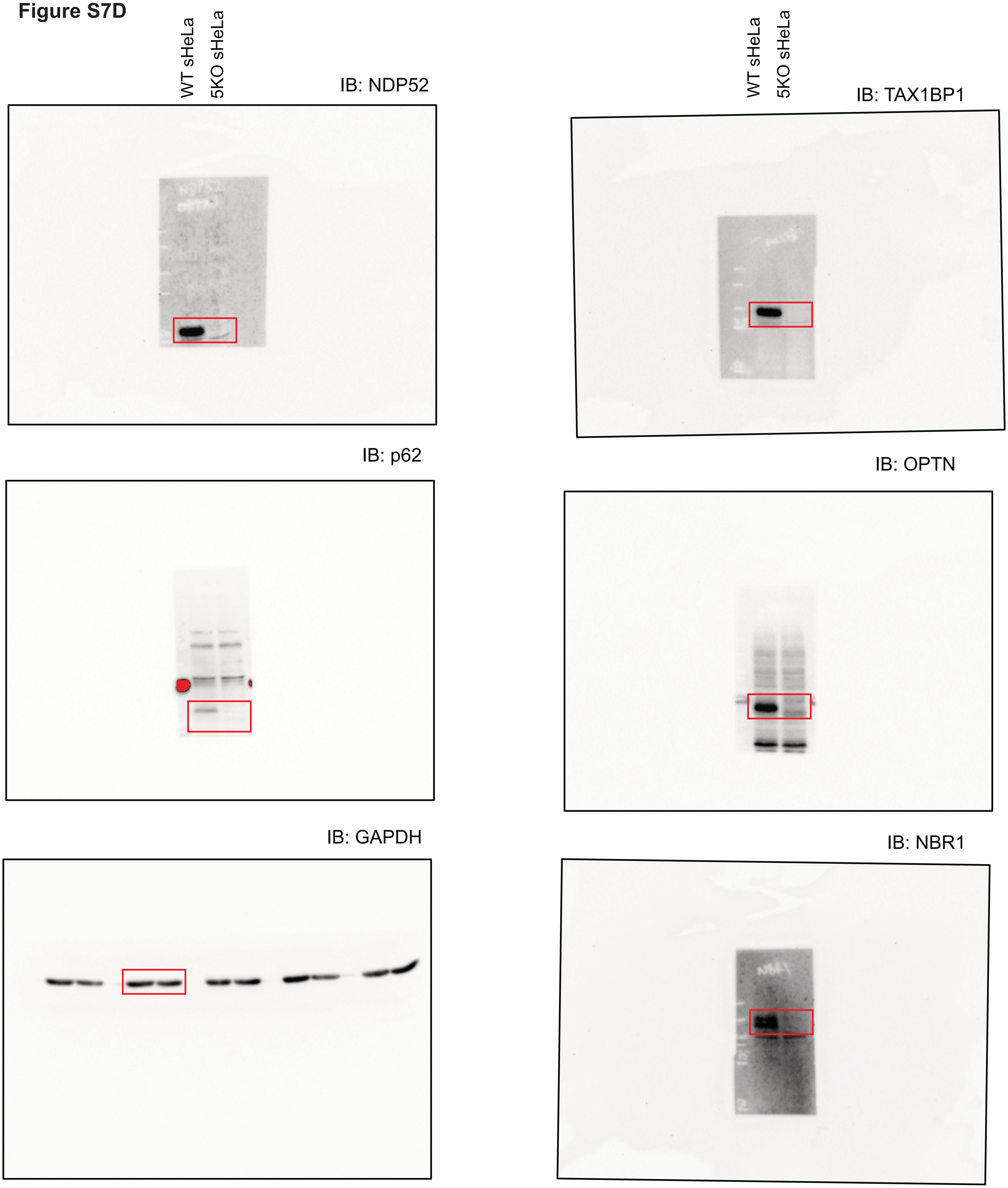
WT sHeLa
5KO sHeLa
IB: NDP52
WT sHeLa
5KO sHeLa
IB: TAX1BP1
IB: p62
IB: OPTN
IB: GAPDH
IB: NBR1
