# Supplementary material for: Exposure of the inner mitochondrial membrane triggers apoptotic mitophagy
Source: Cell Death Differ. 2024 Feb 23;31(3):335–47. doi: 10.1038/s41418-024-01260-2 (PMC10923902; doi:10.1038/s41418-024-01260-2)
Supplement: Supplementary file 16 — Supplementary Movie Legendes [file 41418_2024_1260_MOESM16_ESM.docx]

**Movie 1 Herniating mitochondria are enveloped by LC3B during apoptosis.**

*Mcl1^-/-^* MEFs expressing TOMM20-Halo (stained with JF646, pink), TFAM-mScarlet (yellow), and GFP-LC3B (cyan) were imaged utilising spinning-disk microscopy. Cells were pre-treated with QVD-OPh [20µM] and imaging began after the addition of ABT-737 [1µM].

**Movie 2 Autophagy adaptor P62 is recruited to herniating mitochondria.**

Live-cell spinning disk microscopy imaging of *Mcl1^-/-^* MEFs expressing TOMM20-Halo (stained with JF646) (pink), TFAM-mScarlet (yellow) and, GFP-P62 (dark blue). Cells were pre-treated with QVD-OPh [20µM] and imaging began after the addition of ABT-737 [1µM].

**Movie 3 Autophagy adaptor OPTN is recruited to herniating mitochondria.**

Live-cell spinning disk microscopy imaging of *Mcl1^-/-^* MEFs expressing TOMM20-Halo (stained with JF646) (pink), TFAM-mScarlet (yellow) and GFP-OPTN (blue). Cells were pre-treated with QVD-OPh [20µM] and imaging began after the addition of ABT-737 [1µM].

**Movie 4 Autophagy adaptor NDP52 is recruited to herniating mitochondria.**

Live-cell spinning disk microscopy imaging of *Mcl1^-/-^* MEFs expressing TOMM20-Halo (stained with JF646) (pink), TFAM-mScarlet (yellow) and GFP-NDP52 (pale blue). Cells were pre-treated with QVD-OPh [20µM] and imaging began after the addition of ABT-737 [1µM].

**Movie 5 3D reconstructed tomogram of herniating mitochondria within double-membraned autophagosome.**

Cryo-FIB-tomography revealed a 3D structure of herniating mitochondria being enveloped by an autophagosome.
